# Atg7 in CD4^+^ T cells improves intestinal mucosal inflammation by regulating Ets1‐mediated T cell differentiation

**DOI:** 10.1002/ctm2.70462

**Published:** 2025-08-31

**Authors:** Yue‐Tao Zhou, Quan‐Gui Zhang, Si‐Yuan Du, Hong Chen, Mei‐Zhen Zhu, Min Ai, Shuang‐Shuang Li, Meng‐Nan Dai, Xi‐Ting Wang, Yan Jin, Ying‐Wei Zhu, Jian Lu, Fei Xu, Xue‐Xue Zhu, Li‐Ying Qiu, Hai‐Jian Sun

**Affiliations:** ^1^ MOE Medical Basic Research Innovation Center for Gut Microbiota and Chronic Diseases Wuxi School of Medicine Jiangnan University Wuxi People's Republic of China; ^2^ Wuxi School of Medicine Jiangnan University Wuxi People's Republic of China; ^3^ Laboratory Animal Center Shanghai Jiao Tong University Shanghai People's Republic of China; ^4^ Department of Nursing Shanghai Seventh People's Hospital Shanghai People's Republic of China; ^5^ Department ofGastroenterology Nanjing Medical University Affiliated Wuxi Second Hospital Wuxi People's Republic of China; ^6^ Department of Gastroenterology The First Affiliated Hospital of Fujian Medical University Fuzhou People's Republic of China; ^7^ Department of Gastroenterology Central Hospital Affiliated to Jiangnan University (Wuxi Second People's Hospital) Wuxi People's Republic of China

**Keywords:** Atg7, autophagy, CD4^+^ T cells, IBD, Th1 differentiation

## Abstract

**Background:**

Atg7‐autophagy–related gene 7 contributes as an immune cell function regulator, particularly involved in CD4⁺ T cell response. Nevertheless, the specific contribution of Atg7 in CD4⁺ T cells sensitive immune responses in inflammatory bowel disease (IBD) remains largely unclear. This study explores the functional significance and regulatory mechanisms of CD4⁺ T cell‐specific Atg7 in IBD.

**Methods:**

Real‐Time Quantitative Reverse Transcription PCR (qRT‐PCR), western blotting analysis, flow cytometry and immunohistochemistry were employed to evaluate ATG7 expression in peripheral blood and colonic mucosal biopsies from IBD patients. Peripheral CD4⁺ T cells were transfected with lentivirus vectors encoding either *ATG7* (LV‐*ATG7*) or *ATG7* short hairpin RNA (LV‐sh*ATG7*). Furthermore, mice with a CD4⁺ T cell conditional knockout of *Atg7* (*Atg7^ΔCD4^*) were generated, and CD4^+^ T cells of splenic origin were subjected to RNA‐seq.

**Results:**

*ATG7* level was markedly elevated in inflamed mucosa tissues and peripheral blood CD4^+^ T cells of patients with active Crohn's disease compared to healthy individuals. Overexpression of *ATG7* suppressed Th1 differentiation while enhancing the induction of iTreg cells. *Atg7^ΔCD4^* mice exhibited exacerbated result of 2,4,6‐trinitrobenzenesulphonic acid‐induced experimental colitis, as did *Rag1^−/−^* mice adoptively transferred with CD45^RBhigh^ CD4^+^ T cells from *Atg7^ΔCD4^* donors. RNA‐seq analysis revealed that E26 transformation‐specific sequence‐1 (*Ets1*) acts downstream of *Atg7* and mediates its regulatory effects on Th1 and Treg cell differentiation.

**Conclusion:**

Our data indicate that Atg7 alleviates mucosal inflammation by modulating Th1/Treg differentiation through the regulation of Ets1 expression. Thus, CD4⁺ T cell‐expressed Atg7 may serve as a promising therapeutic approach for IBD.

**Key points:**

- Atg7 expression was significantly elevated in the inflamed mucosa and blood CD4^+^ T cells of patients with active Crohn's disease.
- Specific‐knockout of Atg7 in CD4^+^ T cells leads to exacerbated colitis severity.
- Ets1 functions as a target of Atg7, where it inhibits Th1 cell differentiation and promotes Treg cell differentiation.

## Graphical abstract

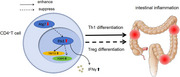

## 1 INTRODUCTION

Crohn's disease and ulcerative colitis (inflammatory bowel disease, IBD), demonstrate chronic intestinal inflammatory process driven by a breakdown in immune tolerance to antigens in the gut lumen.^1^ IBD is a relapsing, inflammatory gastrointestinal condition with complex pathogenesis, involving factors such as immunity, genetics, environment and intestinal microbes.^2^, ^3^ Increasing attention is being given to immune dysfunction in the development of IBD. Mounting evidence points to IBD arises from disrupted mucosal immune homeostasis, leading to altered intestinal flora, activation of pathogenic T cells and chronic inflammation.^4^ Among the various inflammatory cells in the intestine, mucosal CD4^+^ T cells are pivotal in inducing and maintaining chronic inflammation through their production of proinflammatory cytokines. Upon encountering antigen and becoming activated, CD4⁺ T cells diversify into several lineages as Th1, Th2, Th9, Th17, Th22, T follicular helper cell (Tfh) and various regulatory T cell (Treg) populations.^5^ These subsets respond to local microenvironmental cues and participate in intricate interactions with other immune cells, thereby influencing the development process of IBD.

Autophagy is an important physiological and pathological process in which intracellular lysosomes degrade mitochondria and other intracellular components.^6^ ATG proteins are organized into functional modules in mammalian systems, which coordinate autophagosome formation.^7^ Autophagy shows significance in maintaining intestinal homeostasis, with Atg7 acting as a core regulator. As an E1‐like enzyme, Atg7 facilitates the Atg12 and Atg8/LC3 conjugation by ubiquitin‐like system via mediating their interactions with the E2‐like enzymes Atg10 and Atg3.^8^, ^9^ Atg7 acts as an enzyme to catalyse the ATP‐dependent activation of Atg12 and Atg8/LC3 at its catalytic cysteine site, forming thioester intermediates before transferring them to Atg10 and Atg3, respectively.^8^, ^10^ Atg7 mediates the Atg12–Atg5–Atg16L1 complex assembly, which is essential for phagophore membrane expansion.^11^ Additionally, Atg7 processes Atg8/LC3, enabling its C‐terminal cleavage and subsequent phosphatidylethanolamine (PE) conjugation, which is critical for autophagosome maturation and closure.^8^ Beyond its enzymatic function, Atg7 is indispensable for autophagy initiation by coordinating the conjugation of multiple core autophagy proteins.^10^

Dysfunction in Atg7 is associated with various diseases, including amyotrophic lateral sclerosis, neurodegenerative diseases, cancer and several immune system disorders.^12^, ^13^, ^14^ Genome‐wide association studies have identified multiple polymorphic changes in autophagy‐related genes among IBD patients, highlighting the potential significance of autophagy in IBD pathogenesis.^13^, ^14^ Studies have shown that enteroepithelial‐specific *Atg7* gene knockout mice (*Atg7^IEC‐KO^*) exhibited defective granule cell exocytosis and colitis symptoms.^15^ Specific deletion of Atg7 in antigen‐presenting cells (*Atg7^ΔAPC^*) in mice enhances the inflammatory Th17 cell response, causing mitochondrial dysfunction and oxidative stress. Additionally, specific deletion of *Atg7* in myeloid cells may increase susceptibility to colitis.^16^, ^17^ However, the exact role of Atg7 expressed in CD4^+^ T cells in maintaining gut homeostasis during colitis continues to be explored. As a consequence, we studied the role and mechanism of Atg7 in controlling CD4^+^ T cell differentiation as well as sustaining the gut environment balance in colitis mice.

Our results show that ATG7 as a key regulator governing Th1/Treg cell‐mediated intestinal inflammatory responses in patients with CD. The raised level of Atg7 in CD4^+^ T cells from individuals in the active CD (A‐CD) group was found compared to healthy controls (HC). Transfection of peripheral blood CD4^+^ T cells (CD) with LV‐*ATG7* inhibited Th1 differentiation while promoting Treg differentiation. Experiments with *Atg7^ΔCD4^* mice revealed more severe colitis induced by 2,4,6‐trinitrobenzenesulphonic acid (TNBS) enema or by recombination with *Rag1^−/−^* mice using CD45^RBhigh^CD4^+^ T cells. RNA‐seq analysis revealed that *Ets1* functions downstream of *Atg7* and contributes to the regulation of Th1/Treg balancing in IBD. Atg7 facilitated the lysosomal degradation of Ets1 through transferring Ets1 to the lysosome. In vitro experiments culturing splenic naive CD4^+^ T cells from *Atg7^ΔCD4^* mice showed decreased Th1 cytokine levels and increased Treg‐related cytokine levels, as assessed by Real‐Time Quantitative Reverse Transcription PCR (qRT‐PCR). These findings demonstrate that Atg7 directly regulates Th1 and Treg cell differentiation by downregulating Ets1 through the autophagy‐lysosome pathway, thereby ameliorating intestinal mucosal inflammation.

## 2 METHODS

### 2.1 Subjects

Samples for colon biopsies and blood were from IBD patients and healthy control subjects at Wuxi Second People's Hospital, China, between September 2023 and April 2024. The study cohorts consisted of 9 A‐CD patients, 14 active UC (A‐UC) patients and 13 HCs who underwent routine endoscopy examinations. Blood samples were from the A‐CD group (*n* = 9), A‐UC group (*n* = 14) as well as HC group (*n* = 28). The CD or UC is diagnosed based on clinical features, radiographic and endoscopic observations, along with histopathological assessment.

### 2.2 Lentiviral transduction of CD4^+^ T cells

Utilize the anti‐human primary CD4^+^ T cell enrichment kit from Invitrogen to separate CD4^+^ T cells from blood. Employ anti‐CD3 (eBioscience) and anti‐CD28 monoclonal antibodies (eBioscience) in 5 and 2 µg/mL for stimulation in vitro. Cells preactivated (2 × 10^5^ cells/well) were transduced with LV containing LV‐*ATG7*, LV‐sh*ATG7*, and LV‐NC (negative control). Cells were incubated for 5 days, harvested, and assessed for transduction efficiency as well as expression levels via qRT‐PCR.

### 2.3 Mice

Six to eight weeks' male C57BL/6 *Rag1^−/−^* mice were utilized. *Rag1^−/−^*, *Atg7^fl/fl^* and *Atg7^ΔCD4^* mice were housed in a specific pathogen‐free space. Our experiments were taken with approval from Laboratory Animal Management Committee of the School of Medicine, Jiangnan University. *Rag1^−/−^* mice (strain number, T004753) were purchased by Jicui Yaokang Biotechnology Co., LTD. CD4^cre^ mice (strain number, CKOCMS191126JN1‐SUP) were purchased by Saiye Model Biology Research Center (Taicang) Co., LTD. *Atg7^fl/fl^* mice were kindly provided by Professor Li Zhu from Soochow University. *Atg7^ΔCD4^* mice were obtained by hybridizing *Atg7^fl/fl^* mice with CD4^cre^ mice.

### 2.4 siRNA transfection of CD4^+^ T cells

About 2 × 10^5^/well CD4^+^ T lymphocytes were seeded following activation for 12–24 h, and si‐*Ets1* and si‐NC were added and transfected into 1640 culture medium without double antibodies and serum. The mixture was gently mixed under room temperature for 5 min. Subsequently, the transfection diluent was mixed with other diluents at a 1:1 ratio and allowed to sit for 15–20 min. The original culture was removed, washed with phosphate buffer saline (PBS), and replaced with culture medium devoid of double antibodies and serum along with the aforementioned mixture. After 6 h incubation, the culture medium was replaced by the complete cell culture medium for next steps. The siRNA sequence targeting *Ets1* is as follows: 5′‐ GCC GGA CAU GAC CAA AGU ATT ‐3′ (Forward), 5′‐ UAC UGU GGA CAU GAC AGG CTT ‐3′ (Reverse).

### 2.5 Th cell and iTreg cell differentiation in vitro

Two hours prior to the experiment, anti‐CD3 combined with anti‐CD28 antibodies at a ratio of 1:2 was dissolved in sterile PBS. About 50 µL/well of the solution was added in 96‐well plate and incubated at 37°C in a cell culture incubator. After negative isolation of mouse CD4^+^ T cells with magnetic beads (Invitrogen), antibodies were drawn from the plate prepared earlier and washed twice with 200 µL PBS. And 2 × 10^5^/well naive CD4^+^ T cells were seeded followed by cytokines combined with antibodies induction based on the desired differentiation, and the volume in each well was adjusted to 200 µL. The differentiation induction conditions of Th cells were based on our previous procedures. After 72 h, cells were harvested for flow cytometry analysis or qRT‐PCR detection. All cytokines were sourced from Biolegend, and neutralizing antibodies from Bioxcell.

### 2.6 Trinitrobenzenesulphonic‐acid‐induced acute mice colitis

Following a 24‐h fasting period, *Atg7^ΔCD4^* and *Atg7^fl/fl^* mice (6–8‐week‐old) were anaesthetized using 4% chloral hydrate. Subsequently, these anaesthetized mice were intrarectally administered 150 µL of 2.5% TNBS or 50% ethanol in a progressive manner. Monitoring for signs of acute colitis, such as diarrhoea, bloody stools and changes in body weight, was conducted daily. After 7‐day administration, mice were euthanized, and colon tissues were collected for analysis. Colon length was recorded, and tissues were then fixed in 4% polyformaldehyde, embedded in paraffin followed with sectioned, after which haematoxylin and eosin (H&E) staining as well as the immunohistochemical staining were analysed. Histologically score of colonic inflammation was performed by a 0–4 grading scale.^18^

### 2.7 Adoptive T cell transfer‐induced chronic colitis model in *Rag1^−/−^* mice

After negative selection of CD4^+^ T lymphocytes from *Atg7^ΔCD4^* and *Atg7^fl/fl^* mice from spleen using anti‐mouse magnetic beads (Invitrogen), CD25^−^CD45^RBhigh^CD4^+^ T cells were intraperitoneal injected into Rag*^−/−^* mice (5–10 × 10^5^ cells/mouse). Controls were injected with equal volume PBS. Following cell transplantation, mice were monitored for disease over an 8‐week period. Subsequently, mice were euthanized, and H&E staining, immunohistochemistry, flow cytometry and qRT‐PCR were performed.

### 2.8 Isolation of lamina propria mononuclear cells

The colonic fragments were placed in 5 mL of pre‐digestion solution (HBSS without calcium and magnesium, including 5 mM EDTA+5% fetal bovine serum‐FBS) and incubated in a constant‐temperature shaking shaker at 37°C, 250 rpm, for 40 min. The remaining tissue fragments were transferred to a digestion solution (1640 medium, 5% FBS, 0.15 g collagenase IV, 0.025 g DNaseI) preheated to 37°C, and incubated in a constant‐temperature shaking shaker at 37°C, 250 rpm, for 30 min. Lymphocytes were isolated using Percoll separation buffer, resuspended in PBS, and washed twice for subsequent flow cytometry experiments.

### 2.9 Flow cytometric analysis

Isolated CD4⁺ T lymphocytes were activated and incubated at 37°C for 6 h. Surface staining was performed using murine or human antibodies (eBioscience) for CD4⁺ T cells. After fixation and permeabilization with the Foxp3 Fixation/Permeabilization solution (eBioscience), intracellular staining was carried out with antibodies targeting murine or human proteins. Data were then collected for analysis.

### 2.10 Statistical analysis

Mean ± standard deviation (SD) values are presented and were analyzed using GraphPad Prism software (version 10). Based on the experimental design, the following statistical tests were applied: unpaired or paired Student’s t‐tests, multiple t‐tests, one‐way analysis of variance (ANOVA) with Dunnett’s post hoc method, two‐way ANOVA followed by Sidak’s multiple comparisons test, or unpaired Student’s t‐test where appropriate. A p‐value of less than 0.05 was considered statistically significant.

## 3 RESULTS

### 3.1 ATG7 is upregulated in active CD patients

**FIGURE 1:**
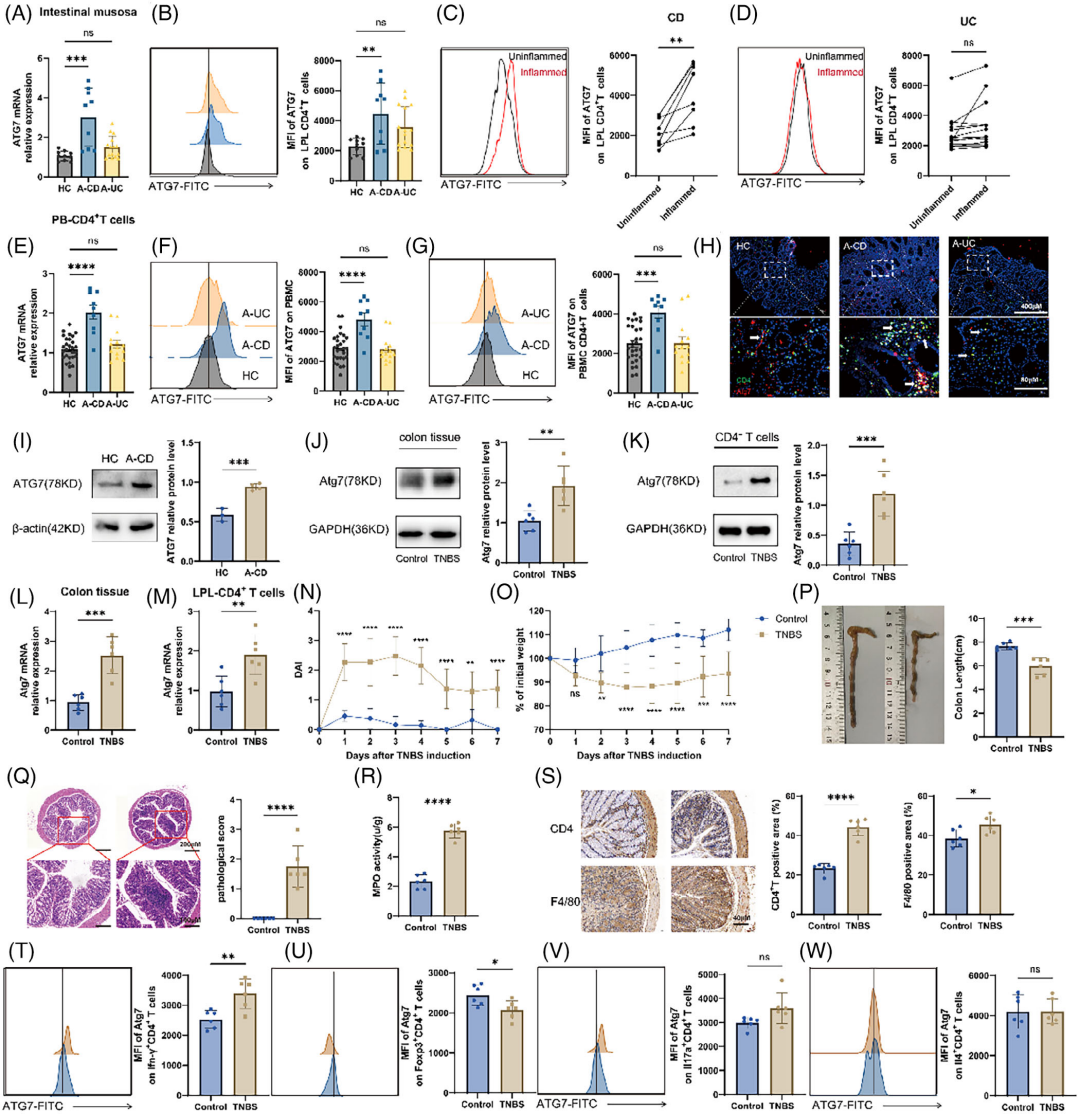
Atg7 is upregulated in active CD patients and colitis mice. (A) The relative expression of *ATG7* mRNA in colon biopsy specimens was investigated using qRT‐PCR in 9 A‐UC, 14 A‐CD and 13 HC. (B) Lamina propria lymphocytes (LPLs)‐CD4^+^ T cells were collected from 9 A‐UC, 14 A‐CD and 13 HC. Median fluorescence intensity (MFI) of intracellular ATG7 expression was detected by flow cytometry. (C) ATG7 expression in matched inflamed and uninflamed colon biopsy specimens of 9 A‐CD patients. (D) ATG7 expression in matched inflamed and uninflamed colon biopsy specimens of 14 A‐UC patients. (E) The relative levels of *ATG7* mRNA were assessed using qRT‐PCR from 9 A‐CD, 14 A‐UC and 28 HC in PB‐CD4^+^ T cells. (F) ATG7 expression was assessed in PBMC cells from 28 HC, 9 A‐CD and 14 A‐UC using flow cytometry. (G) ATG7 expression in peripheral blood CD4^+^ T cells was analysed on samples from 28 HC, 9 A‐CD and 14 A‐UC individuals using flow cytometry. (H) Immunofluorescence double staining of ATG7 with CD4 was observed through in samples from HC, A‐CD and A‐UC groups for co‐localization confirmation. (I) The ATG7 protein level was assessed in human intestinal samples. (J, K) Western blotting (WB) was applied to assess the protein level of Atg7 in mouse colon tissue and LPL‐CD4^+^ T cells. (L, M) The mRNA expression of Atg7 in both mouse colon and LPL‐CD4^+^ T cells was detected. (N) Daily assessment of disease activity index (DAI) was applied after 2,4,6‐trinitrobenzenesulphonic acid (TNBS) treatment. (O) Daily body weight of mice after TNBS administration. (P) Changes in the appearance of the colon and colon length of dissected mice on day 7. (Q) Histopathological features and scoring of TNBS‐induced acute colitis in mice (H&E staining). Scale bars, 200 µm. (R) Assessment of myeloperoxidase (MPO) activity in a model of TNBS‐induced acute colitis. (S) Representative 40‐µm‐scale images showing CD4⁺ T cells and F4/80⁺ macrophages. The proportion of positive cells was calculated using ImageJ. (T–W) The expression of Atg7 in Ifn‐γ^+^ CD4^+^, Foxp3^+^ CD4^+^, Il17a^+^ CD4^+^ and Il4^+^ CD4^+^ T cells of mouse colon LPL was assessed. *n* = 6 in each group for J–W. Bars represent the mean ± SD from multiple independent experiments. Significance was determined by one‐way analysis of variance (ANOVA) with Dunnett's method or the unpaired Student's *t* test where applicable. ns, *p *> .05, *p < .05,**p < .01, ***p < .001, ****p < .0001.

The expression profile of ATG7 was analysed in individuals suffering from IBD to test its contribution in the development of IBD. ATG7 expression was assessed through qRT‐PCR and flow cytometry analysis (Figure 1A–D). Quantitative analysis revealed significantly elevated ATG7 mRNA expression and protein levels (as measured by MFI) in A‐CD patients versus HC, whereas A‐UC patients showed no statistically significant differences (Figure 1A,B). Interestingly, a higher ATG7 protein level was detected in the inflamed mucosa of the same CD patient when compared to the non‐inflamed mucosa (Figure 1C,D). Moreover, analysis of peripheral blood mononuclear cells (PBMC) cells and PB‐CD4^+^ T revealed an upregulation of ATG7 expression in A‐CD patients relative to controls (Figure 1E–G). Higher ATG7 abundance in CD4^+^ T cells from A‐CD patients was showed in the immunofluorescence double staining (Figure 1H). In line with this, the protein expression of ATG7 tended to be higher in human intestinal samples (Figure 1I). To validate these results, colitis was induced in wild mice using TNBS as an experimental model. The TNBS mice had higher Atg7 expression than the untreated group, particularly in CD4^+^ T cells, consistent with clinical data (Figure 1J,K). The mRNA level of *Atg7* in both mouse colon and CD4⁺ T lymphocytes isolated from colonic lamina propria lymphocytes (LPLs) was also elevated in TNBS mice (Figure 1L,M). Compared with control mice, TNBS‐induced mice had higher DAI score and lower body weight (Figure 1N,O). In addition, TNBS mice exhibited shortened colon length and increased colon pathological score (Figure 1P,Q). The activity of myeloperoxidase (MPO), CD4^+^ cell expression and macrophages were upregulated in the colon of TNBS mice versus healthy mice (Figure 1R,S). Further assessment through flow cytometry demonstrated that the elevated Atg7 levels in CD4^+^ T cells were primarily linked to an augmentation in Atg7 expression in Th1 cells (Figure 1T–W). Consistently, the transcriptional level of ATG7 was elevated in IBD patients from the database of GSE119600 and GSE94648 (Figure S1). Besides, ATG7 was upregulated in CD4^+^ T cells at inflammatory loci in patients with colitis from single cell sequencing database (GSE226875) (Figure S2). Notably, flow cytometric data analysis revealed that the expression of ATG7 in peripheral blood CD8⁺, CD14⁺, CD19⁺ and CD11c⁺ cells had no significant difference between healthy individuals and IBD patients (Figure S3).

### 3.2 Suppression of ATG7 in CD4^+^ T cells enhances Th1 cell development and suppresses Treg cells generation in CD4^+^ T cells of A‐CD patients

**FIGURE 2:**
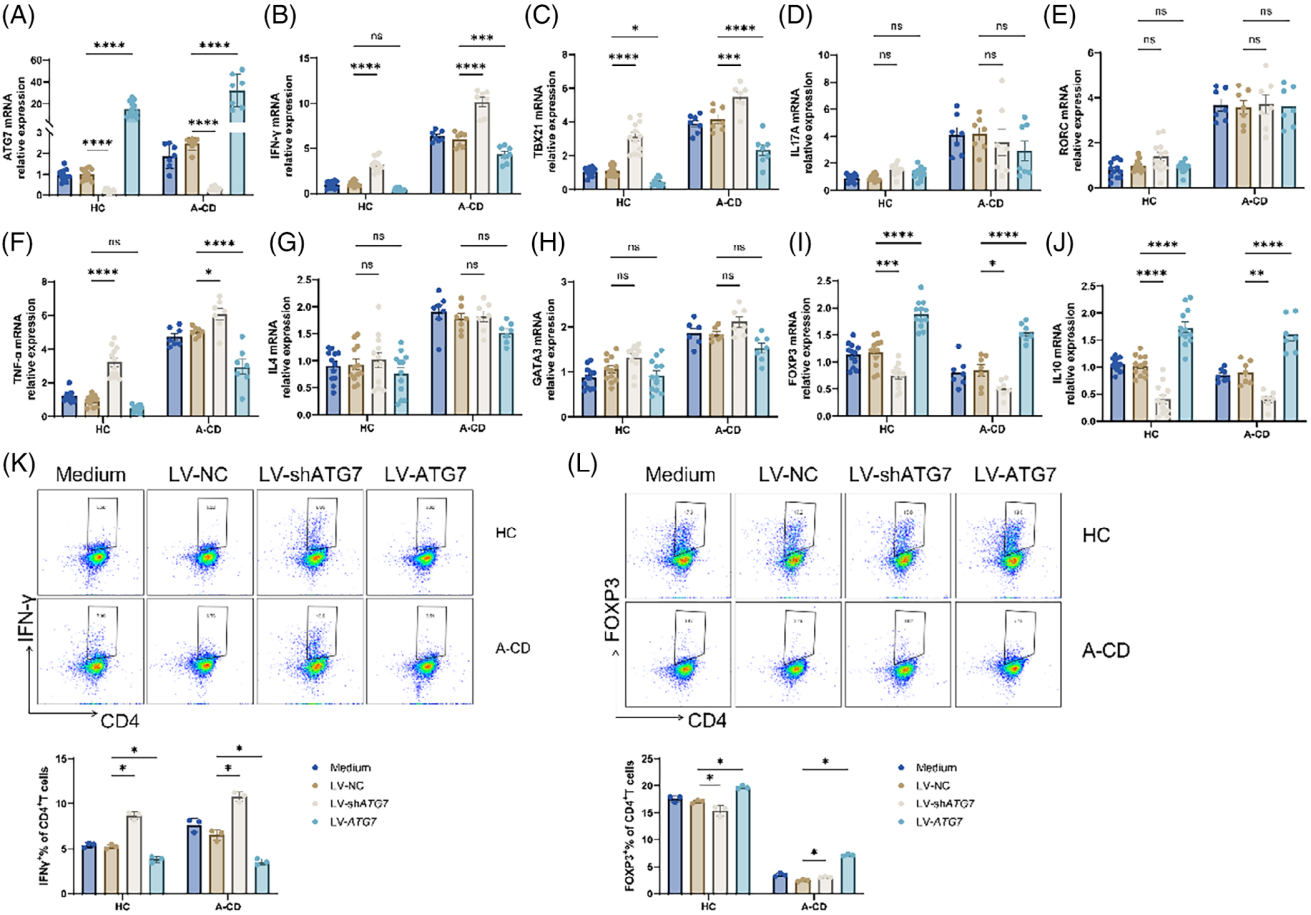
Suppression of *ATG7* enhances Th1 cell development and suppresses Treg cells generation in CD4^+^ T cells of A‐CD patients. (A–L) 12 HC and 7 A‐CD individuals PB‐CD4^+^ T cells underwent transfection with lentiviruses that express LV‐sh*ATG7*, LV‐*ATG7* and negative control. Taking 5 days to incubate cells with coated 5 µg/mL anti‐CD3 antibody combined soluble 2 µg/mL anti‐CD28 antibody for 5 days. (A) On day 5, CD4^+^ T cells that had been transfected were gathered, and validation of LV‐sh*ATG7* and LV‐*ATG7* transduction efficiency though qRT‐PCR. (B–J) qRT‐PCR was used to detect the *IFN‐γ*, *TBX21*, *IL17A*, *RORC*, *TNF‐α*, *IL4*, *GATA3*, *FOXP3* and *IL10* expression in the CD4^+^ T cells that were transfected. The data are normalized to the HC medium for A–J. (K, L) IFN‐γ^+^CD4^+^ and FOXP3^+^CD4^+^ T cells were detected via flow cytometry and the cells percentage in figure. Bars represent the mean ± SD from multiple independent experiments. Two‐way analysis of variance (ANOVA) with Sidak's test. ns (*p *> .05), *p < .05,**p < .01, ***p < .001, ****p < .0001.

The increase in ATG7 expression in A‐CD patients prompted our investigation into its effects on the differentiation of CD4^+^ T cell. Subsequently, we transfected purified CD4^+^ T cell with LV‐ATG7, LV‐sh*ATG7* and a negative control (NC) in both healthy control and active CD patient groups. Our results demonstrated a noteworthy rise in *ATG7* expression at mRNA and protein levels in CD4^+^ T cells transfected with LV‐*ATG7* relative to the control (LV‐NC), while LV‐sh*ATG7* transfection effectively diminished *ATG7* (Figure 2A and Figure S4). Further examination of cytokine and transcription factor expression showed that A‐CD individuals had reduced amounts of *IFN‐γ*, *TNF‐α* and *TBX21* but increased *FOXP3* and *IL10* levels in LV‐*ATG7*‐transfected CD4^+^ T cells (Figure 2B,C,F,I,J). In addition, blocking *ATG7* in CD4^+^ T cells from A‐CD led to contrasting results. Importantly, no significant variances were noted in *IL4*, *GATA3*, *IL17A* and *RORC* expression levels (Figure 2D,E,G,H). Upon re‐examination of our data and relevant literatures, we recognize that the A‐CD group may represent a unique immunological context where compensatory or feedback mechanisms, possibly linked to chronic immune dysregulation or differential metabolic states, alter the involvement of autophagy in Treg cells development (Figure 2L). The results of flow cytometry analysis verified the effect of ATG7 knockout or overexpression on the Th1/ Treg differentiation (Figure 2K,L).

### 3.3 *Atg7* deficiency has opposing effects on Th1 and Treg cell differentiation

**FIGURE 3:**
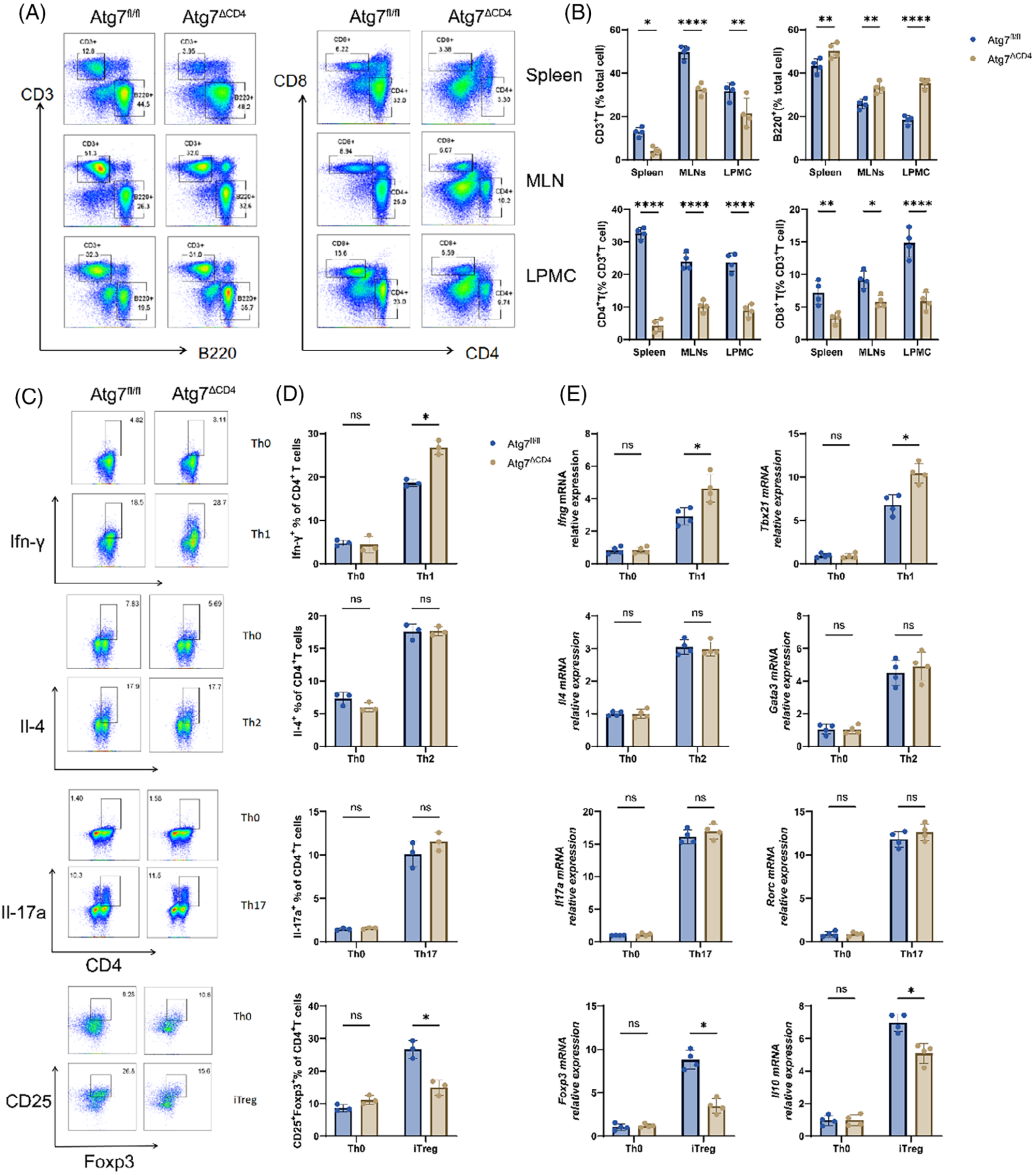
Atg7 deficiency has opposing effects on Th1 and Treg cell differentiation. (A)Utilize flow cytometry to detect B220^+^ cells, CD3^+^ T cells, CD4^+^ T and CD8^+^ T cells in spleen, mesenteric lymph node (MLN) and lamina propria mononuclear cell (LPMC). (B) The percentage of cells in (A) was counted. (C) Detection of mouse CD4^+^ T cell differentiation using flow cytometry. (D) Statistical analysis of lineage differentiation of CD4⁺ T cell in mouse. (E) Detection of mouse CD4^+^ T cell differentiation by qRT‐PCR. Bars represent the mean ± SD from multiple independent experiments. Two‐way ANOVA with Sidak's test. ns (*p *> .05), *p < .05,**p < .01, ****p < .0001.

To explore impacts of *Atg7* on Th1/Treg differentiation balance and its effect on inflammation of intestinal mucosa, CD4^+^ T cell‐specific *Atg7* knockout mice (*Atg7^ΔCD4^*) were generated (Figure S5). No significant difference was observed in the body weight, spleen index, colon length, and the histological staining of liver, kidney, lung, heart and spleen between wild‐type mice and CD4^+^ T cell‐specific *Atg7* knockout mice (Figure S6). Our research demonstrated that deleting *Atg7* led to reduced proportion of CD3^+^ T cells along with CD4^+^ and CD8^+^ populations, with a concurrent increase in B220^+^ cells levels in the spleen, mesenteric lymph node (MLN) and lamina propria mononuclear cell (LPMC compared to *Atg7^fl/fl^* mice, indicating compromised immune function (Figure 3A,B). Naïve CD4^+^ T cells with a purity of 90% were activated though co‐stimulation with anti‐CD3 and anti‐CD28 and cultured followed with different induction conditions. Demonstrating hallmark activation phenotypes (Figure S7). Flow cytometry analysis after 3 days showed alterations in differentiation patterns of Th1, Th2, Th17 and Treg cells in *Atg7^ΔCD4^* and *Atg7^fl/fl^* mice. *Atg7^ΔCD4^* naive T cells showed an elevation in Th1 cell population and a reduction in Treg cells, with no significant differences in Th2 and Th17 cell differentiation (Figure 3C,D and Figure S8). These observations were validated through relevant cytokines and transcription factors expression profiles assessed by qRT‐PCR (Figure 3E). CFSE staining demonstrated that *Atg7* deletion inhibited the overall proliferation of CD4⁺ T cells (Figure S9A,B). However, *Atg7* deficiency differentially modulated Th and Treg cell proliferation depending on polarization conditions: it enhanced Th1 cells proliferation, while suppressing Treg cells proliferation (Figure S9A,B). No significant differences were observed on Th2 or Th17 cells (Figure S9A,B).

### 3.4 Deficiency of Atg7 in CD4^+^ T cells exacerbates TNBS‐induced colitis in mice

**FIGURE 4:**
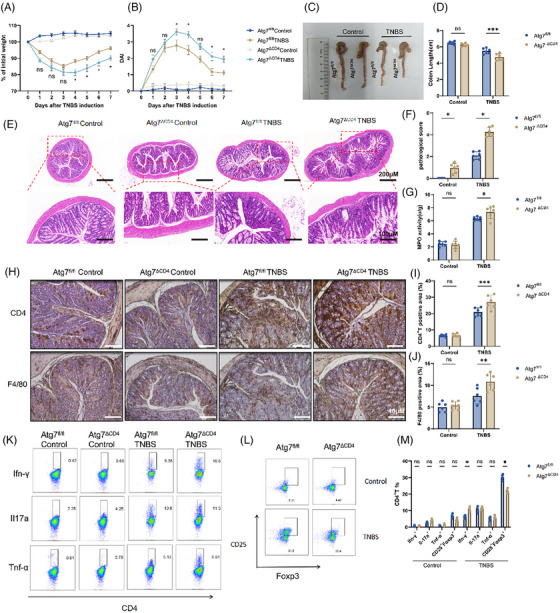
Deficiency of Atg7 in CD4^+^ T cells exacerbates colitis in mice. (A) Daily body weight of mice after 2,4,6‐trinitrobenzenesulphonic acid (TNBS) administration. (B) Daily assessment of DAI after TNBS administration. (C) Changes in the appearance of the colon on day 7. (D) Changes in colon length of dissected mice on day 7. (E) Establishment of representative colon sections of TNBS‐induced acute colitis in *Atg7^ΔCD4^* and *Atg7^fl/fl^* mice with H&E staining. Scale bars, 200 and 100 µm. (F) Establishment of histopathological scores of colonic tissues. (G) Comparison of myeloperoxidase (MPO) activity in a TNBS‐induced acute colitis. (H) Representative 40‐µm‐scale images showing CD4⁺ T cells and F4/80⁺ macrophages. (I, J) The proportion of positive cells was calculated using ImageJ. (K) The expressions of Ifn‐γ, Il‐17a and Tnf‐α in LPL‐CD4^+^ T cells after 6 h stimulation with phorbol 12‐myristate 13‐acetate (PMA), ionomycin along with last 2 h Brefeldin A were analysed using flow cytometry. (L) CD25 and Foxp3 expression of LPL‐CD4^+^ T cells was assessed via flow cytometry. (M) The proportion of Ifn‐γ^+^CD4^+^ T, Il‐17a^+^CD4^+^ T, Tnf‐α^+^CD4^+^ T, CD25^+^Foxp3^+^CD4^+^ T. *n* = 6/group. Bars stand for the mean ± SD from multiple independent experiments. Two‐way analysis of variance (ANOVA) with Sidak's test. Compared with the *Atg7^fl/fl^* CD4^+^ T cell group (A, B). ns (*p *> .05), *p < .05,**p < .01, ***p < .001.

For purpose of validating these results, a mouse model of colitis induced by TNBS was created. As a result, *Atg7^ΔCD4^* mice exhibited loss of body weight, sever disease activity index values, shortened colon segments, increased pathological values and increased MPO activity compared with *Atg7^fl/fl^* littermate control mice (Figure 4A–G). Atg7 deficiency in CD4^+^ T cells promoted the abundance of both CD4^+^ T cells and macrophages in the colon of mice (Figure 4H–J). Flow cytometry analysis revealed elevated expressing Ifn‐γ and Tnf‐α expressing in CD4^+^ T cells, along with decreased levels of CD25 and Foxp3, within the LPLs of *Atg7^ΔCD4^* mice as compared to TNBS‐induced *Atg7^fl/fl^* controls (Figure 4K–M). Consistently, the transcript levels of *Ifn‐γ*, *Tbx21* and *Tnf‐α* were elevated while *Foxp3* and *Il10* levels were decreased of TNBS‐induced *Atg7^ΔCD4^* colitis mice relative to littermate controls born with *Atg7^fl/fl^* (Figure S10). Notably, no differences were found in levels of *Il17a*, *Rorc*, *Il4* and *Gata3* (Figure S10). In summary, these findings suggested that deficiency of *Atg7* worsens TNBS‐induced colitis by enhancing Th1 immune responses and hindering Treg cell development.

### 3.5 *Atg7^ΔCD4^* CD4^+^ T cells induce more severe colitis in *Rag1^−/−^* mice

**FIGURE 5:**
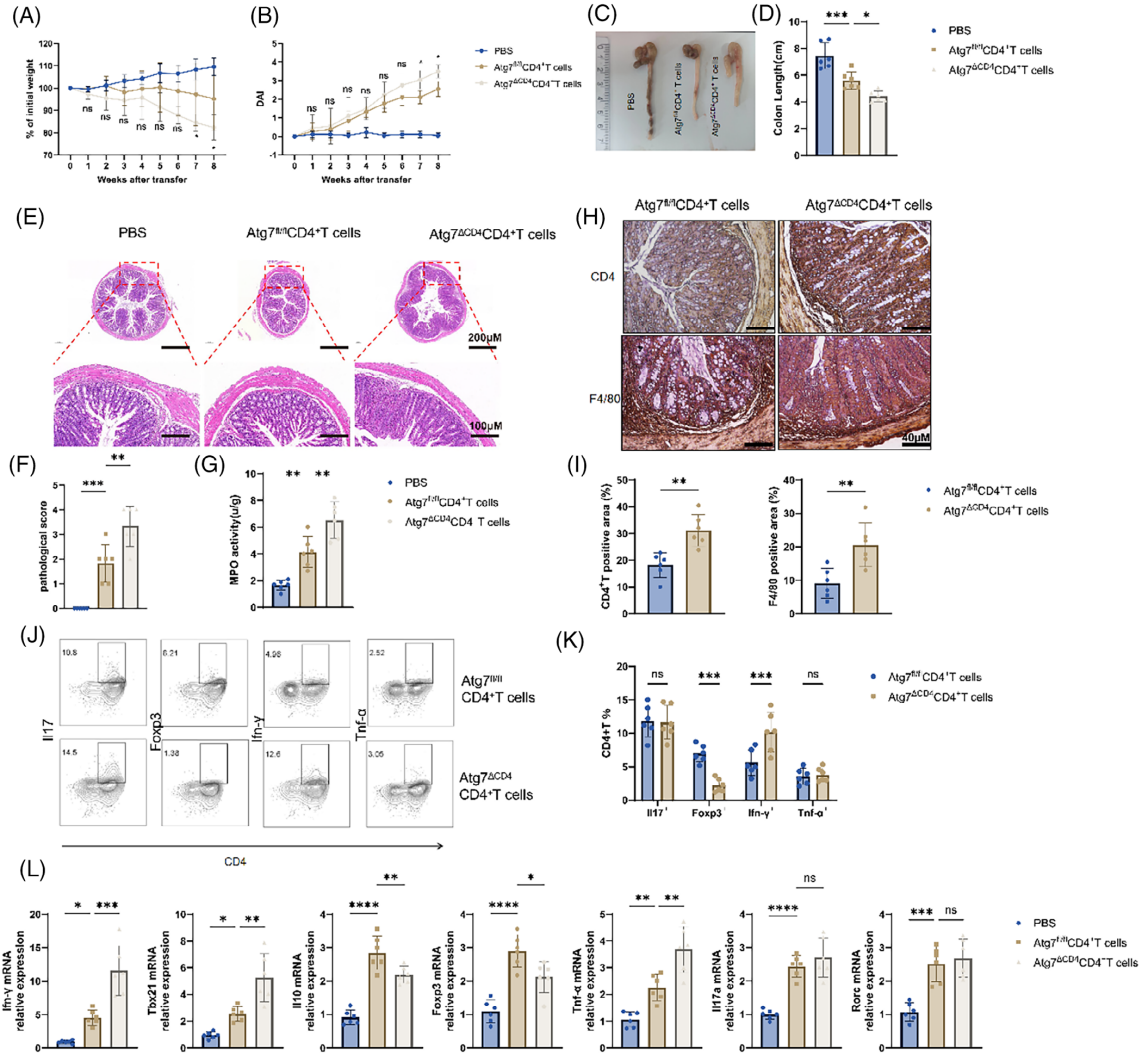
Atg7 deficiency in CD4^+^ T cells leads to more severe colitis in *Rag1^−/−^* mice. (A) Changes in body mass index. (B) Disease activity index. (C) Colon appearance. (D) Colon length. (E) Representative colon sections with H&E staining. (F) Histopathological score. (G) myeloperoxidase (MPO) neutrophil activity value. (H) Immunohistochemical staining of representative colon sections. (I) positive percentage of CD4^+^ T cells and F4/80 cells. (J, K) Flow cytometry of *Atg7^ΔCD4^* CD4^+^ T and *Atg7^fl/fl^* CD4^+^ T cell adoptive *Rag1^−/−^* mice to construct LPL CD4^+^ T cells in mice with chronic colitis. (L) qRT‐PCR detection of cytokine expression of *Atg7^ΔCD4^* CD4^+^ T and *Atg7^fl/fl^* CD4^+^ T cell adoptive *Rag1^−/−^* mice constructed from mouse chronic colitis. Bars represent the mean ± SD derived from repeated independent replicates. One‐way analysis of variance (ANOVA) with Dunnett's method or the unpaired Student's *t* test. Two‐way NOVA with Sidak's test. Compared with the *Atg7^fl/fl^* CD4^+^ T cell group (A, B). Each group *n* = 6. ns (*p *> .05), *p < .05,**p < .01, ***p < .001, ****p < .0001.

To confirm Atg7's inhibitory effect in the differentiation of Th1 cell and its promotion differentiation of Treg cell, our study delved into the potential of *Atg7^ΔCD4^* CD4^+^ T cells in exacerbating gut inflammation. To investigate this, we utilized a chronic colitis CD4^+^ T cell transfer model in *Rag1^−/−^* mice. Specifically, *Atg7^ΔCD4^* and *Atg7^fl/fl^* splenic CD25^−^CD45^RBhigh^CD4^+^ T cells were transferred into *Rag1^−/−^* mice intraperitoneally. Nonetheless, the results shown that *Rag1^−/−^* mice repopulated with *Atg7^ΔCD4^* CD4^+^ T cells exhibited more pronounced colitis symptoms, including greater weight reduction, shorter colon length, higher disease activity index, pathological scores and MPO activity, in contrast to mice repopulated with *Atg7^fl/fl^* CD4^+^ T cells (Figure 5A–G). A noteworthy rise in CD4^+^ T cells and F4/80 macrophages infiltration was evident in the intestinal mucosa, as shown by quantitative statistics of positive areas (Figure 5H, I). Flow cytometry analysis following the isolation of intestinal LPLs revealed more CD4^+^ T cells expressing Ifn‐γ^+^ and Tnf‐α^+^ in *Rag1^−/−^* mice receiving *Atg7^ΔCD4^* CD4^+^ T cells transfer compared to controls. Additionally, decreased percentage of Foxp3^+^CD4^+^ T cells were found (Figure 5J, K). The qRT‐PCR analysis of colon tissue showed similar trends, with higher mRNA levels of *Ifn‐γ*, *Tbx21* and *Tnf‐α*, and lower levels of *Il10* and *Foxp3* in *Rag1^−/−^* mice that received *Atg7^ΔCD4^* CD4^+^ T cells relative to the *Rag1^−/−^* mice that accepted *Atg7^fl/fl^* CD4^+^ T cells (Figure 5L). The deletion of *Atg7* in CD4^+^ T cells exacerbates intestinal inflammatory responses in *Rag1^−/−^* mice by promoting pro‐inflammatory effector Th1 cells and reducing anti‐inflammatory Treg cells.

### 3.6 Atg7 plays a role in restricting Th1 immune responses and promoting Treg cell immune responses through the regulation of Ets1

**FIGURE 6:**
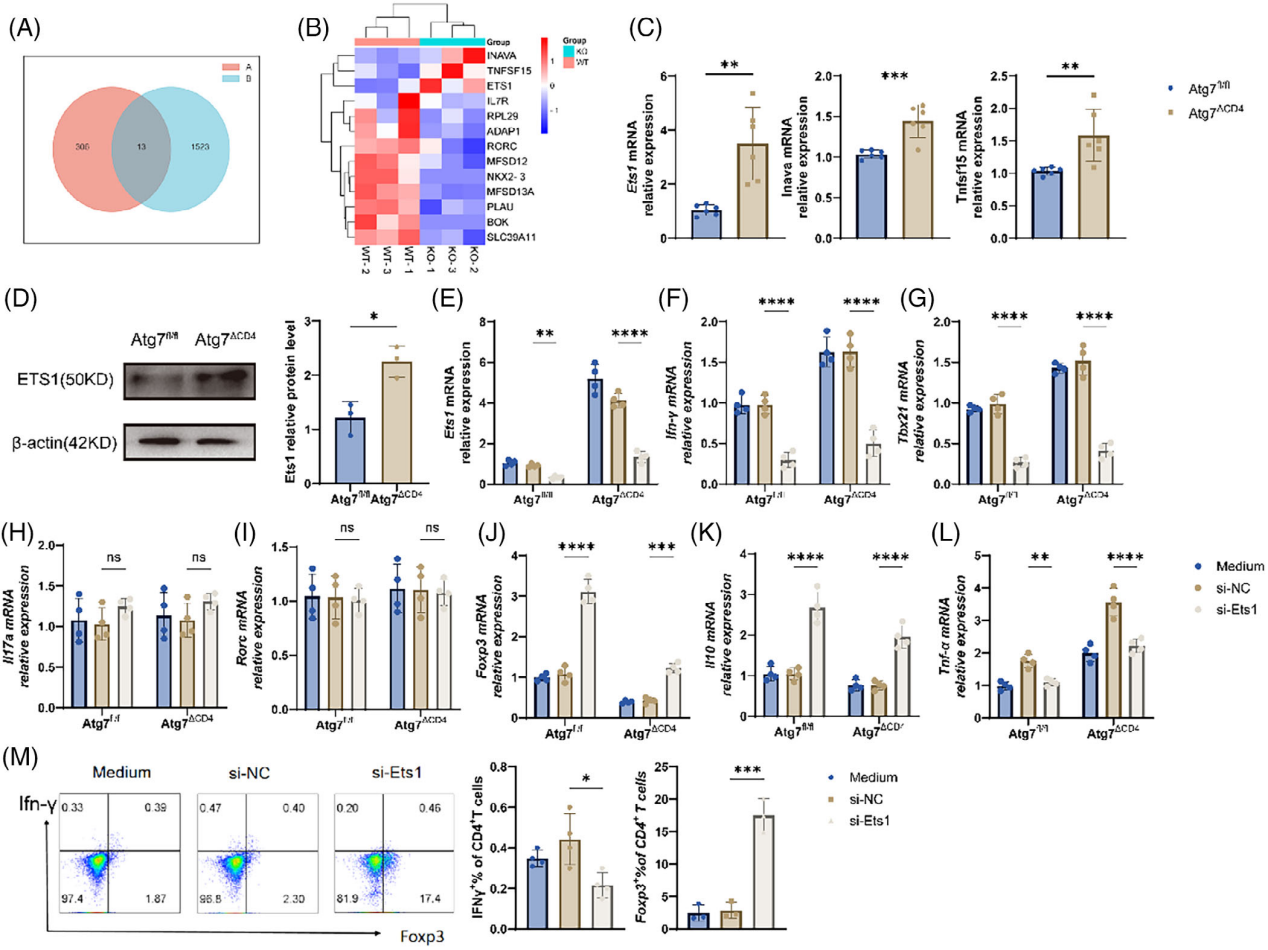
Atg7 promotes Th1 and limits Treg cell immune response by regulating Ets1. (A) Wayne diagram, where A is a significantly different gene by RNA sequencing and B is an IBD susceptibility gene, and the intersection is taken. (B) Heat map, based on clustering of 13 overlapping genes. (C) The expression of *Ets1, Inava* and *Tnfsf15* at the mRNA levels in CD4^+^ T cells of *Atg7^ΔCD4^* and *Atg7^fl/fl^* mice. (D) The expression of Ets1 at the protein level in *Atg7^ΔCD4^* and *Atg7^fl/fl^* CD4^+^ T cells. (E) The transfection efficiency of si‐*Ets1* was examined by qRT‐PCR. (F–L) Splenic naive *Atg7^fl/fl^* and *Atg7^ΔCD4^* CD4^+^ T cells were transfected with si‐*Ets1* to detect cytokine and transcription factor expression via qRT‐PCR, respectively. (M) Flow cytometry analysis of percentages of Ifn‐γ^+^ and Foxp3^+^ from *Atg7^ΔCD4^* CD4^+^ T cells activated after knockdown of *Ets1*. Bars stand for the mean ± SD derived from repeated independent replicates. One‐way analysis of variance (ANOVA) with Dunnett's method or the unpaired Student's *t* test. Two‐way NOVA with Sidak's test. ns (*p *> .05), *p < .05,**p < .01, ***p < .001, ****p < .0001.

To delve deeper into the molecular mechanism through which Atg7 enhances colitis and controls the CD4^+^ T cells functions, we obtained naive CD4^+^ T cells derived from the spleen of *Atg7^ΔCD4^* and *Atg7^fl/fl^* mice. The differential expression genes of cells were then analysed at the transcriptional level using RNA seq transcriptomic sequencing. The overlap between genes with differential expression and IBD‐related susceptibility genes between the two groups was used to screen out the molecule *Inava*, *Ets1* and *Tnfsf15* (Figure 6A,B). *Inava*, *Ets1* and *Tnfsf15* mRNA levels were markedly increased in CD4^+^ T cells of *Atg7^ΔCD4^* mice, with the highest increase detected in *Ets1* (Figure 6C). *Ets1* is extensively expressed in various kinds of immune cells, playing a crucial part in T cell functionality.^18^, ^19^ Studies have indicated that *Ets1* is notably elevated in individuals with IBD and contributes to the elevation of Th1 cells. We found higher Ets1 expression of CD4^+^ T cells of *Atg7^ΔCD4^* at mRNA and protein levels when compared with *Atg7^fl/fl^* mice (Figure 6C,D), which was consistent with the transcriptomic sequencing. *Ets1* siRNA (si‐*Ets1*) was then used to transfect primary *Atg7^ΔCD4^* and *Atg7^fl/fl^* CD4^+^ T cells. Silencing *Ets1* prevented the differentiation of Th1 cells and facilitated the differentiation of Treg cells, as evidenced by the measurement of cytokine and transcription factor expression of transcripts (Figure 6E–L). Importantly, flow cytometry analysis further demonstrated that downregulation of *Ets1* impeded Th1 cell differentiation and promoted Treg cell polarization (Figure 6M). These findings suggest that *Atg7* might influence dynamic balance of Th1 and Treg differentiation in IBD by modulating *Ets1*.

In light of the role of Atg7 in the process of autophagy, we examined whether ATG7 modulated Ets1 levels through pathways associated with autophagy‐mediated protein degradation. To investigate the effects of Atg7 deletion on the autophagy/lysosome pathway, we performed LysoTracker staining and immunofluorescence staining for the lysosomal marker LC3b. LysoTracker staining revealed that, lower fluorescence intensity was observed in naive CD4^+^ T cells from *Atg7^ΔCD4^* mice compared to those from *Atg7^fl/fl^* mice, suggesting that Atg7 deletion leads to decreased lysosomal number and activity (Figure S11A). Immunofluorescence analysis further showed that LC3b fluorescence intensity was decreased, while Ets1 fluorescence intensity was increased in *Atg7^ΔCD4^* mice. Moreover, the colocalization of LC3b and Ets1 was reduced, suggesting that *Atg7* deletion may impair the lysosomal degradation of Ets1 (Figure S11B). These results suggest that Atg7 is essential for maintaining normal lysosomal function in CD4⁺ T cells. When Atg7 is deleted, the number and activity of lysosomes decrease, leading to impaired autophagy. The reduced colocalization of LC3b and Ets1 further supports the idea that Atg7 facilitates the lysosomal degradation of Ets1. Therefore, loss of Atg7 disrupts the degradation of Ets1, which may contribute to abnormal T cell differentiation and promote inflammation, effects that were observed in IBD. To investigate whether Atg7 regulates Ets1 expression through the autophagy pathway, CD4^+^ T cells were purified from *Atg7^ΔCD4^* mice and induced autophagy using rapamycin, a known autophagy inducer. Subsequently, the cells were collected for immunofluorescence analysis, which showed a marked reduction in Ets1 fluorescence intensity in the rapamycin‐treated group (Figure S12). These findings further substantiate that ATG7 modulates ETS1 expression via the autophagy pathway.

### 3.7 Atg7 might regulate the intestinal microbiota in mice

The intestinal microbiota primarily involved in IBD pathogenesis. Dysbiosis, characterized by an imbalance in microbial composition, has been closely associated with immune responses dysregulation and persistent intestinal inflammatory processes in IBD patients. It is interesting to know whether there is a change in the intestinal microbiota between *Atg7^ΔCD4^* and *Atg7^fl/fl^* mice. We thus conducted the 16s rDNA sequencing experiment in mice faeces. Clear group separation was found via principal coordinate analysis and non‐metric multidimensional scaling (NMDS) (Figure S13A), indicating that Atg7 deletion altered the gut microbiota diversity in mice. Compared with *Atg7^fl/fl^* mice, *Atg7^ΔCD4^* mice exhibited a decrease in the observed species index and an increase in the Shannon diversity index, suggesting a reduction in microbial richness but an increase in microbial uncertainty or diversity (Figure S13B,C). At a variety of taxonomic ranks, including phylum, class, order, family, genus and species, the dominant gut microbiota in mice belonged to the phyla Bacteroidota and Firmicutes. Compared to the *Atg7^fl/fl^* mice, the *Atg7^ΔCD4^* mice showed greater Bacteroidota prevalence and a lower relative abundance of Firmicutes. The Firmicutes/Bacteroidota (F/B) ratio was reduced in the *Atg7^ΔCD4^* mice (Figure S13D), which was consistent with previous findings that patients with IBD exhibiting a decreased F/B ratio.^21^ Phylogenetic Investigation of Communities by Reconstruction of Unobserved States (PICRUSt2) data demonstrated significant enrichment of cell growth and death, signal networks, carbohydrate and amino acid metabolism pathways in the gut microbiome of *Atg7^ΔCD4^* mice compared to *Atg7^fl/fl^* mice (Figure S13E). These results suggest that Atg7 in CD4⁺ T cells is important for maintaining gut microbiota homeostasis. Its deletion leads to microbial dysbiosis characterized by reduced richness, altered composition, and an imbalance between Firmicutes and Bacteroidota, changes associated with increased susceptibility to IBD‐like inflammation. Future studies are warranted to investigate the mechanistic pathways through which Atg7‐deficient T cells influence gut microbial communities, including possible alterations in cytokine production, mucosal barrier function and microbial metabolite profiles.

## 4 DISCUSSION

Abnormal CD4^+^ T cell activation, their infiltration and dysfunction in the colon are key characteristics of IBD. It is believed that CD4^+^ T cell‐mediated inflammation acts a key part in the IBD development and experimental colitis.^22^, ^23^ A comprehensive understanding of the pivotal regulators governing T lymphocyte activation and differentiation is imperative for the IBD treatment.^24^, ^25^, ^26^ Our research has revealed that Atg7 acts a key part in slowing down the symptoms of IBD by inhibiting Th1 reactions and enhancing Treg cell‐driven immune responses. It is worth noting that Atg7 has been linked to the development of lung, thyroid, and triple‐negative breast cancer.^27^, ^28^, ^29^, ^30^, ^31^ Furthermore, diminished Atg7 protein expression has been observed in PBMCs from multiple sclerosis patients and spinal cords from animals with autoimmune encephalomyelitis, suggesting the involvement of Atg7 in immune‐related disorders.^32^, ^33^ However, the exact contribution of Atg7 to the development of IBD is still unclear. In this investigation, a notable rise in levels of ATG7 was detected in the inflamed mucosa as well as in PB‐CD4^+^ T cells among A‐CD individuals. Additionally, it is noteworthy that ATG7 is up‐regulated in A‐CD patients and experimental colitis mouse models, predominantly expressed in pro‐inflammatory CD4^+^IFNγ^+^ T cells, indicating that ATG7 affects the CD4^+^ T cells differentiation in the process of inflammation. Previous research has shown that the knockout of *Atg7* in intestinal epithelial cells causes to elevated levels of pro‐inflammatory genes and heightened inflammatory responses to endotoxin.^34^, ^35^, ^36^ Atg7 knockout mice exhibit increased susceptibility to *Citrobacter rodentium* infection, resulting in heightened disease severity, possibly because of impaired lipid accumulation product (LAP).^37^ In mice lacking Atg7 in intestinal antigen‐presenting cells, there is an augmented production of mitochondrial reactive oxygen species (ROS) and Th17 inflammation.^16^, ^38^, ^39^ The study postulate that the upregulation of ATG7 in inflamed mucosa of A‐CD patients may be due to CD4^+^ T cell activation and multiple inflammatory cytokines.

Atg7 acts a vital part in regulating CD4^+^ T cell by mediating cytokine production, differentiation and proliferation, through lysosomal degradation of cytoplasmic material.^40^, ^41^, ^42^, ^43^, ^44^ However, in IBD cases, the influence of ATG7 upon the CD4^+^ T cell differentiations remains elusive. For our study, lentiviral transduction experiments were conducted in vitro, revealing that LV‐sh*ATG7* transduction in A‐CD CD4^+^ T cells facilitated differentiations of Th1 cells and suppressed differentiations of Treg cells, while *ATG7* overexpression had the opposite effect. These findings suggest that Atg7 acts on colitis through elastic modulating the immune response of Th1 and Treg cells, which is consistent with earlier studies on the pathogenicity of autophagy regulating CD4^+^ T cell differentiation.^42^, ^45^, ^46^ Additionally, it is noteworthy that Atg7, as a gene related to mitophagosome formation, has been reported to be impaired by ethanol, leading to increased Th1 and decreased Treg differentiation.^47^

To elucidate the influence of Atg7 in CD4^+^ T cell immune responses of mucosal and colitis development, we generated *Atg7* gene CD4^+^ T cell‐specific knockout mice (*Atg7^ΔCD4^* mice) and examined the role of Atg7 on regulating differentiation of CD4^+^ T cell during colitis. CD4^+^ T cell‐specific deficiency of Atg7 disrupted the B220^+^/CD3^+^ cell balance, reflecting immune dysregulation. The study demonstrates that *Atg7* deficiency results in a substantial depletion of memory B cells and concomitant reduction in autoantibody production.^48^ Furthermore, T cell‐derived CD40 ligand (CD40L) mediates B‐cell proliferation through CD40 engagement.^49^ These findings suggest that *Atg7* deficiency may also modulate B‐cell abundance through T cell‐dependent mechanisms. Our findings suggested that *Atg7^ΔCD4^* CD4^+^ T cells had increased Ifn‐γ expression and decreased Foxp3 levels in a Th1 and Treg differentiation context compared to controls. Notably, both *Atg7^ΔCD4^* mice that induced by TNBS rectal administration and *Atg7^ΔCD4^* CD45^RBhigh^CD4^+^ T cell‐reconstituted *Rag1^−/−^* mice, displayed more severe colitis, suggesting that Atg7 deficiency disrupts Th1/Treg cell‐mediated mucosal immunity. The findings are consistent with prior research showing that mice lacking Atg7 specifically in myeloid cells are at increased risk for experimental colitis. This is evidenced by elevated levels of colonic cytokines, a shift towards Th1 polarization, and enhanced systemic bacterial penetration.^17^ Notably, *Rag1^−/−^* mice repopulated with *Atg7^ΔCD4^* CD4^+^ T exhibited elevated *Il17a* and *Rorc* expression, indicating enhanced Th17 cells differentiation. This aligns with prior findings that miR‐146a‐5p promotes Th17 differentiation through Atg7 downregulation.^50^ Paradoxically, while CD4^+^ T cell‐specific *Atg7* deficiency exacerbated colitis, ATG7 expression was upregulated in active IBD patients. We hypothesize this may represent a compensatory response to intestinal inflammation and fibrosis, though the precise mechanistic basis warrants further investigation.

To gain further insight into how Atg7 regulates Th1/Treg cell development, splenic CD4^+^ T cells were isolated from mice, followed by RNA‐seq analysis. Our findings revealed a significantly higher Ets1 expression in *Atg7^ΔCD4^* CD4^+^ T cells than *Atg7^fl/fl^* CD4^+^ T cells. Ets1 can influence CD4^+^ T cell differentiation via Tbx21 or Foxp3.^51^ Extensive genetic studies have pinpointed Ets1 as a vulnerable region for systemic lupus erythematosus and rheumatoid arthritis.^52^ Lack of Ets1 causes systemic lupus erythematosus (SLE) autoimmunity, correlating with the uncontrolled proliferation of T follicular helper type 2 cells.^53^ Because Ets1 has the capacity to coordinate Th17 cell differentiation, which is related to the rheumatoid arthritis (RA) onset.^54^ As an oncogene, Ets1 acts in colorectal cancer, breast cancer, and hepatocellular carcinoma by targeting A variety of microRNAs (such as miR‐125p and miR‐139‐5p), providing evidence that Ets1 is important in tumorigenesis.^19^, ^55^, ^56^, ^57^, ^58^ A recent genetic study also revealed that Ets1 is a susceptibility gene for IBD.^59^ Moreover, Ets1 is crucial in CD4^+^ Th1 cell differentiation. Furthermore, mice deficient in Ets1‐SE exhibit resistance to colitis induced by Th1 cells.^60^ In addition, Ets1 was also noted to act in the maturation of murine CD4^+^ T lymphocytes, and act as a direct target of miR‐125a.^61^ IBD patients have high expression of Ets1, which promotes mucosal inflammation caused by Th1 signalling via cold‐inducible RNA‐binding protein (CIRBP).^20^ Importantly, we reveal a unique mechanism by which Atg7 affects Th1 and Treg cell differentiation by promoting Ets1 expression.

## 5 CONCLUSION

In our study, we found that Atg7 deletion in CD4⁺ T cells aggravated colitis, suggesting the ATG7 functions as protective factor in T cell‐mediated immune regulation. However, the observed upregulation of ATG7 expression in active IBD patients may reflect a compensatory response to heightened inflammatory stress, rather than a direct pathogenic role. In the inflammatory microenvironment of IBD, increased ATG7 expression could be a cellular attempt to counteract excessive immune activation and maintain homeostasis. Moreover, the elevated ATG7 levels in bulk tissues from IBD patients may originate from diverse types of cells beyond CD4⁺ T cells, including epithelial and myeloid cells, where autophagy might be activated to cope with inflammation‐induced stress. Our research indicates that the Atg7 is highly expressed in inflammatory mucosa and blood CD4^+^ T cells in A‐CD. Atg7 affects differentiations of Th1 and Treg in IBD by regulating Ets1.These results underscore the significant impact of Atg7 on IBD development and propose a novel approach targeting Atg7 expression for IBD treatment.

## AUTHOR CONTRIBUTIONS

Yue‐tao Zhou conceived and designed the study. Hong Chen, Mei‐zhen Zhu, Quan‐gui Zhang, and Si‐yuan Du performed the experiments and analyzed the results. Yue‐tao Zhou, Hai‐Jian Sun, Hong Chen, Mei‐zhen Zhu, and Quan‐gui Zhang analyzed and interpreted the data, and drafted the manuscript. Si‐yuan Du, Min Ai, Shuang‐shuang Li, Fei Xu, Xue‐xue Zhu, and Li‐ying Qiu assisted in data interpretation. Xi‐ting Wang, Meng‐nan Dai, Ying‐wei Zhu, Yan Jin and Jian Lu were responsible for patient enrollment and maintenance of the clinical dataset. All authors reviewed and approved the final version of the manuscript.

## CONFLICT OF INTEREST STATEMENT

The authors declare no conflicts of interest.

## ETHICS STATEMENT

Ethical approval was obtained from Medical Ethics Committee of Jiangnan University (JNU202403RB003)  

## Supporting information



Supporting Information

## Data Availability

The data that support the findings of this study are available from the corresponding author upon request.
