# Supplementary material for: Atg7 in CD4+ T cells improves intestinal mucosal inflammation by regulating Ets1‐mediated T cell differentiation
Source: Clin Transl Med. 2025 Aug 31;15(9):e70462. doi: 10.1002/ctm2.70462 (PMC12399787; doi:10.1002/ctm2.70462)
Supplement: Supplementary file 1 — Supporting Information [file CTM2-15-e70462-s001.docx]

**Atg7 in CD4^+^ T cells improves intestinal mucosal inflammation by regulating Ets1-mediated T cell differentiation**

Yue-tao Zhou^a,1*^, Quan-gui Zhang^a,1^, Si-yuan Du^b,1^, Hong Chen^a,1^, Mei-zhen Zhu^b^, Min Ai^c^, Shuang-shuang Li^d^, Meng-nan Dai^e^, Xi-ting Wang^f^, Yan Jin^g^, Ying-wei Zhu^g^ Jian Lu^g^, Fei Xu^b^, Xue-xue Zhu^b^, Li-ying Qiu^b^, Hai-Jian Sun^a*^

^a^MOE Medical Basic Research Innovation Center for Gut Microbiota and Chronic Diseases, Wuxi School of Medicine, Jiangnan University, Wuxi, 214122, PR China.

^b^Wuxi School of Medicine, Jiangnan University, Wuxi, 214122, PR China.

^c^Laboratory Animal Center of Shanghai Jiao Tong University, Shanghai, PR China.

^d^Shanghai Seventh People's Hospital, Shanghai, PR China.

^e^Nanjing Medical University, Nanjing, PR China.

^f^Fujian Medical University, Fuzhou, PR China.

^g^central Hospital affiliated to Jiangnan University (Wuxi second people's Hospital), PR China.

^1^These authors contributed equally to this work (co-first authorship)

^*^Addresses for corresponding authors

Contact information:

Yue-tao Zhou, PhD.

Wuxi Medical School, Jiangnan University

Wuxi 214122, Jiangsu Province

PR China

E-mail addresses: yuetaozhou@126.com

Hai-jian Sun, PhD.

Wuxi Medical School, Jiangnan University

Wuxi 214122, Jiangsu Province

PR China

E-mail addresses: [haijsunjiangnan@jiangnan.edu.cn](mailto:haijsunjiangnan@jiangnan.edu.cn)

**Supplementary Figure**


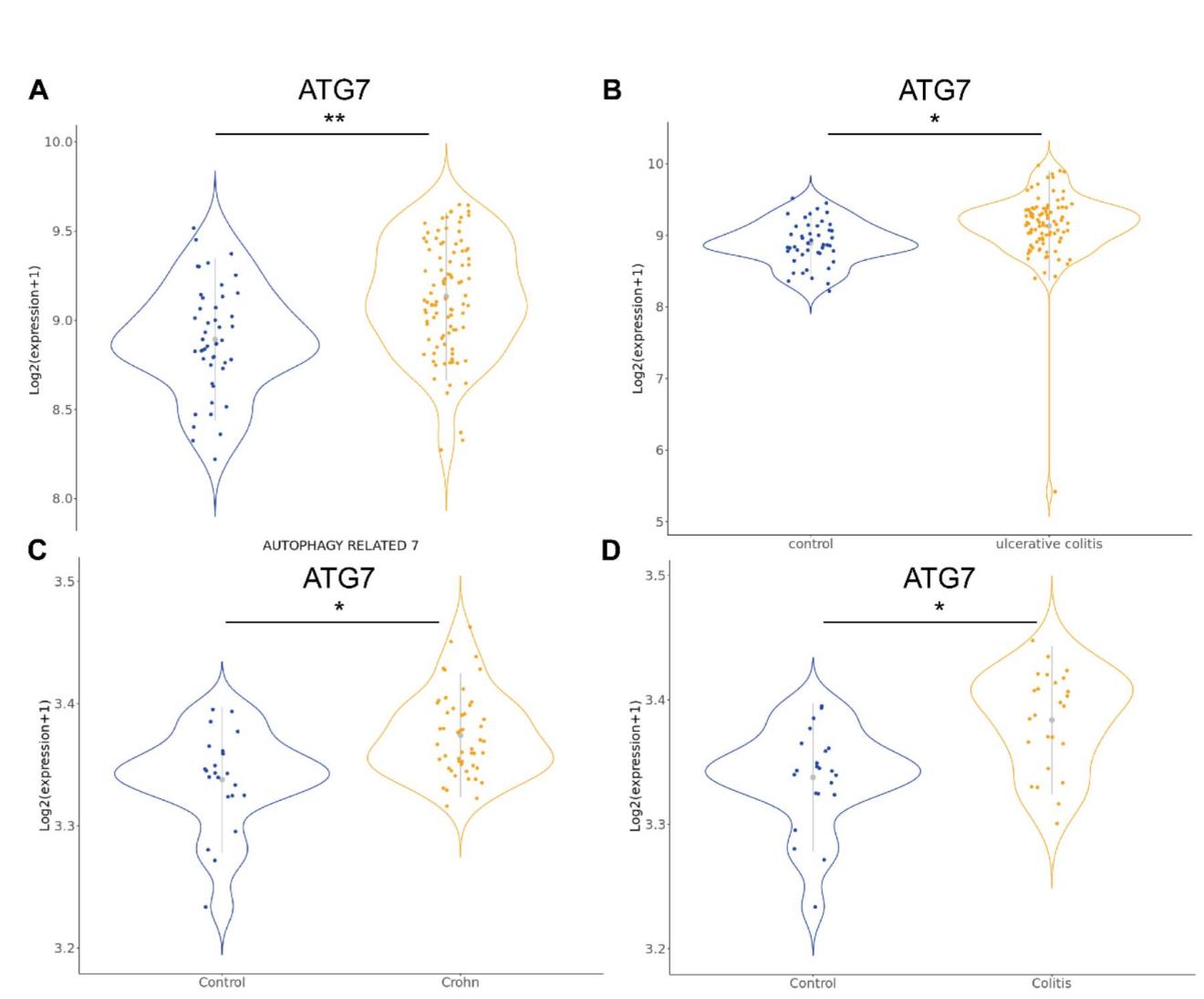


**Figure S1. Atg7 is upregulated in IBD Patients.** (A-D) The expression of Atg7 mRNA in intestinal mucosal biopsy specimen was investigated using the GEO database (GSE119600 and GSE94648). ns, *p*>0.05, **p<*0.05, ***p<*0.01.


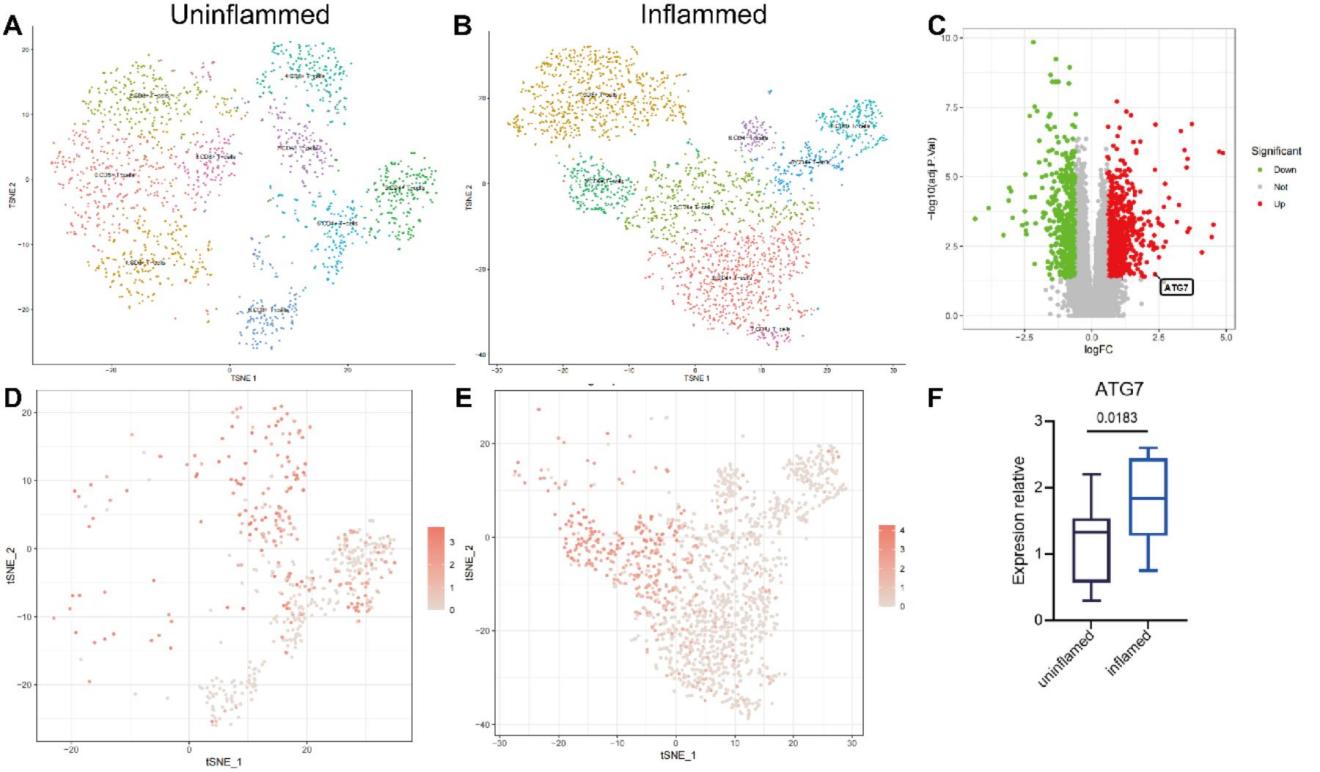


**Figure S2. Atg7 is upregulated in IBD Patients.** (A-D) The expression of Atg7 mRNA in intestinal mucosal biopsy specimen was investigated using the GEO database (GSE119600 and GSE94648)


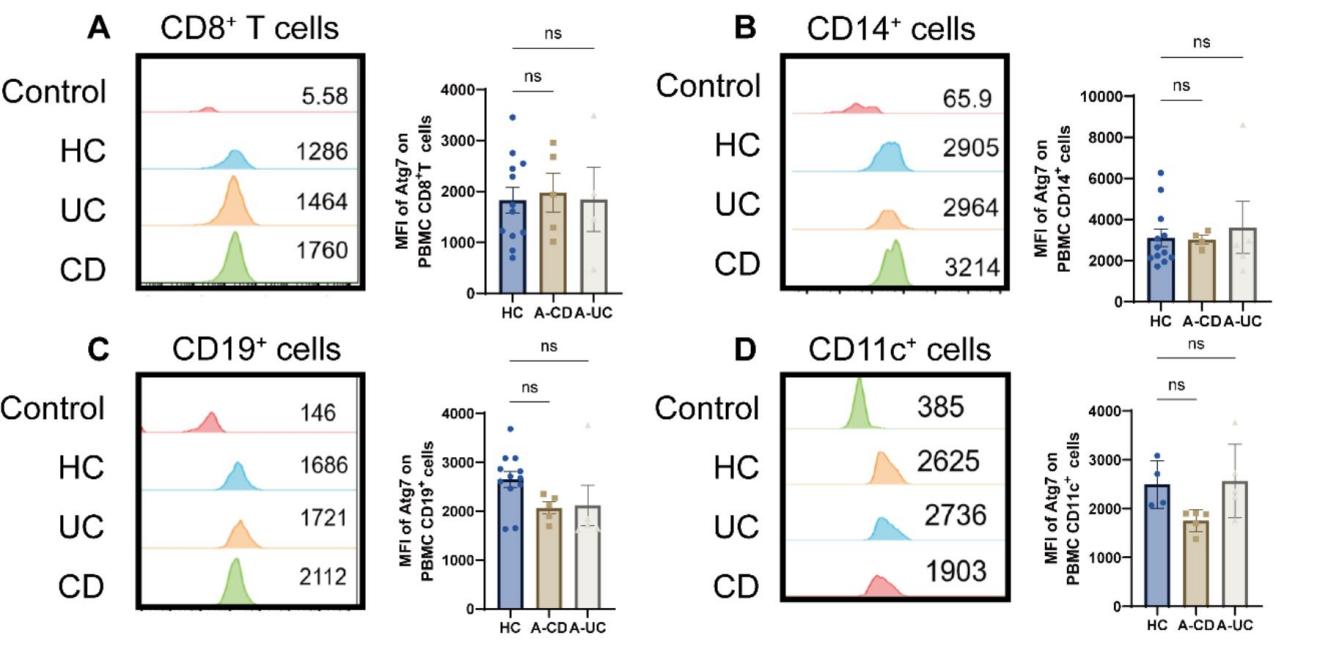


**Figure S3. The expression of** **Atg7 showed no significant difference in other immune cells.** (A-D) Intracellular expression of Atg7 in PB-CD8+ T cells, PB-CD14+ T cells, PB-CD19+T cells and PB-CD11c+ T cells was performed on samples from HC, A-CD, and A-UC individuals using flow cytometry. MFI: Median Fluorescence Intensity. Bars represent the mean ± SD from multiple independent experiments. One-way ANOVA with Dunnett’s multiple comparisons test or the unpaired Student’s t test. ns, p>0.05.


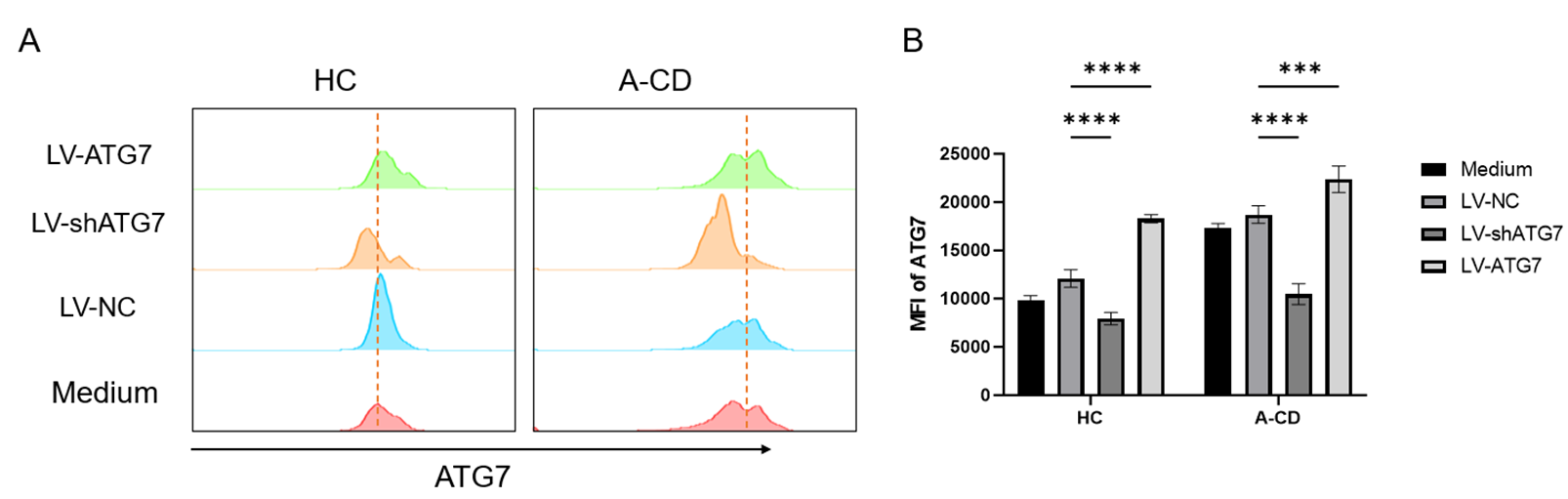


**Figure S4. Protein evaluation following Atg7 transfection.** (A-B) PB-CD4+T cell from 5 HC and 5 A-CD were transfected with lentivirus expressing LV-sh*ATG7*, LV-*ATG7* and negative control. Cells were incubated with immobilized anti-CD3 monoclonal antibody (5μg/ml) and soluble anti-CD28 monoclonal antibody (2μg/ml) for 5 days. On day 5, CD4+ T cells that had been transfected were gathered, and validation of LV-sh*ATG7* and LV-*ATG7* transduction efficiency by flow cytometry. Bars represent the mean ± SD from multiple independent experiments. Two-way ANOVA with Sidak’s multiple comparisons test. ns, *p*>0.05, **p<*0.05, ***p**<*0.01, ****p<*0.001, *****p<*0.0001.
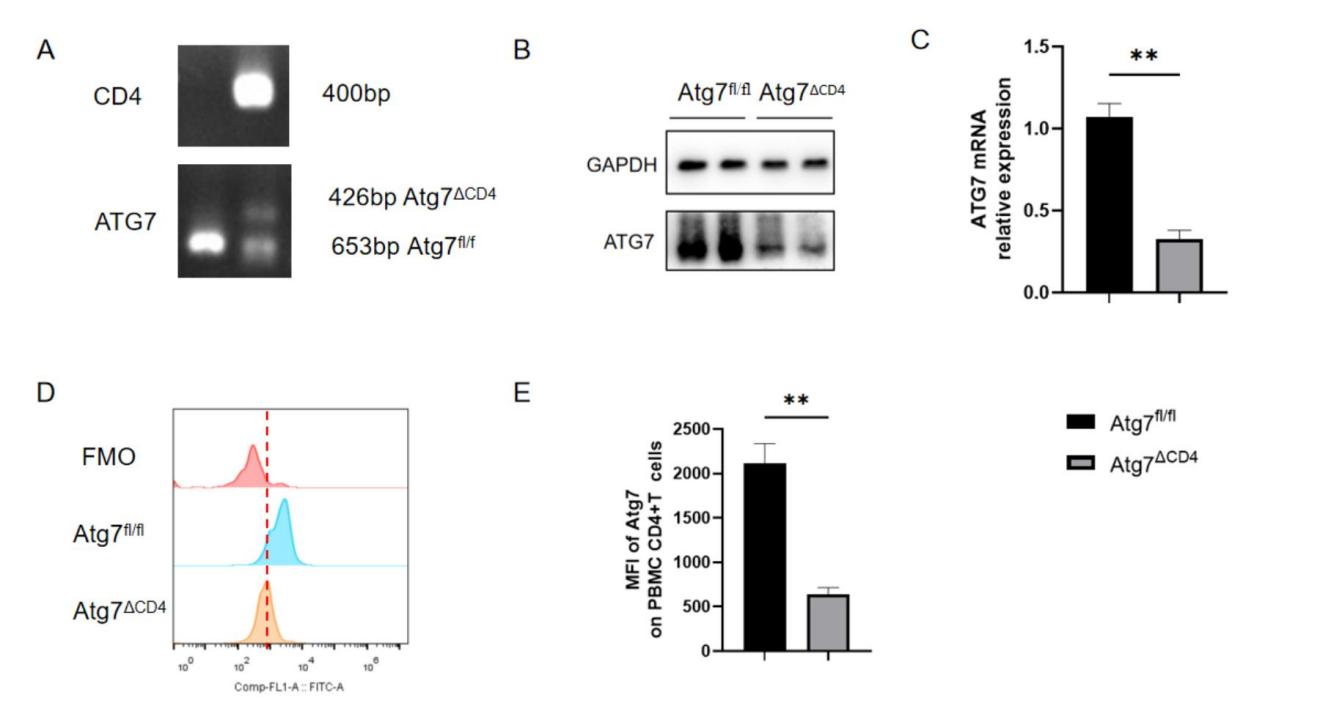


**Figure S5. Validation of CD4**+ **T cell-specific knockout** **Atg7 mice.** (A) Representative images of DNA agarose gel electrophoresis results of Atg7 flox gene and CD4-cre gene. (B) Western blot for protein expression of Atg7. (C) qPCR analysis for the expression of Atg7. (D-E) The average fluorescence intensity of Atg7 expression was analyzed by flow cytometry. n=6 in each group. Bars represent the mean ± SD from multiple independent experiments. One-way ANOVA with Dunnett’s multiple comparisons test or the unpaired Student’s t test. ns, *p*>0.05, **p*<0.05, ***p*<0.01.


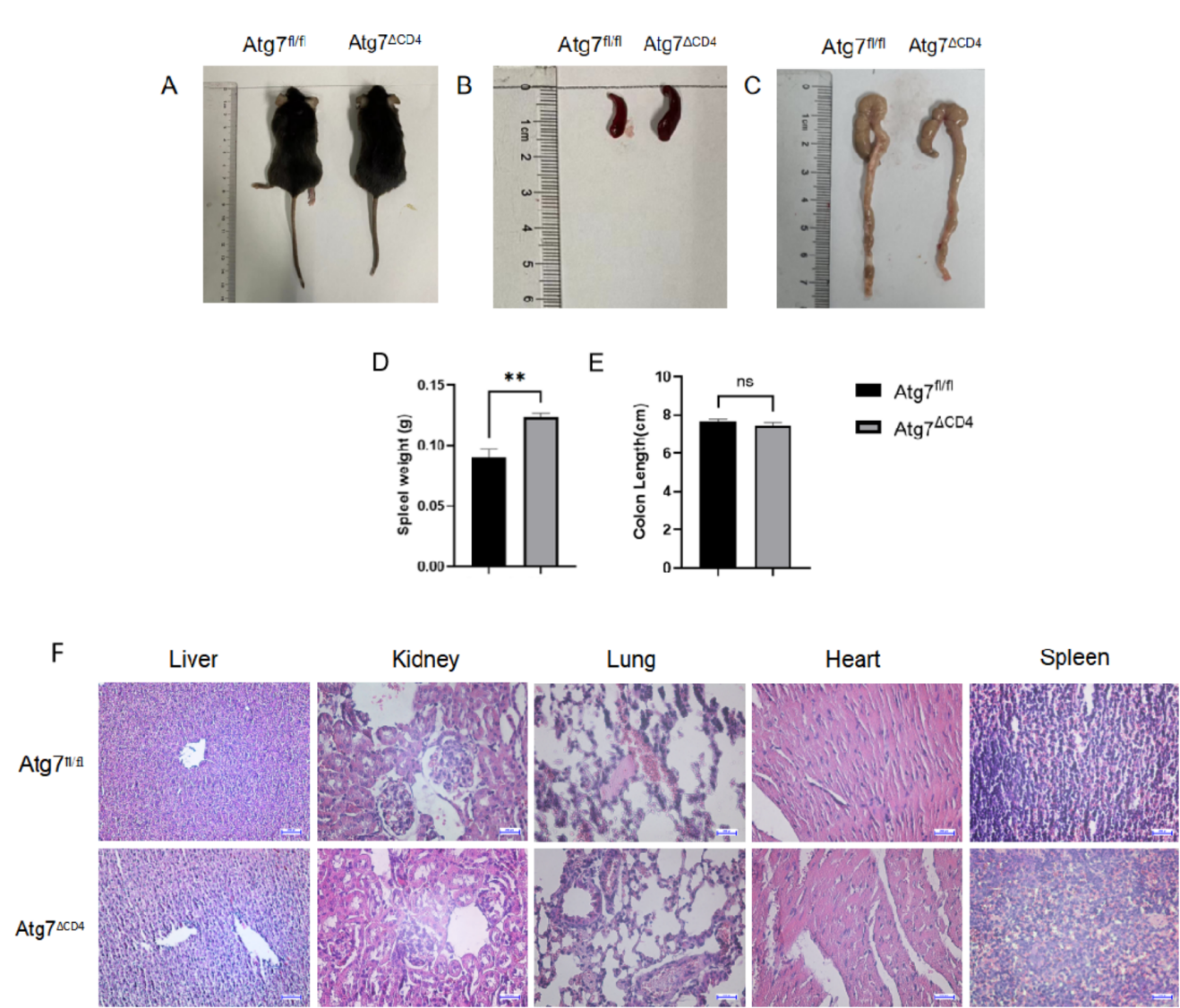

**Figure S6. Differences between *Atg7^fl/fl^*** **mice and** ***Atg7******ΔCD4* mice.** (A-C) Representative image of mouse body size, spleen, and colon. (D-E) Statistical analysis of spleen weight and colon length in mice. (F) H&E staining analysis of liver, kidney, lung, heart, spleen and other important organs of mice. Scale bars, 200µm. n=6 in each group. Bars represent the mean ± SD from multiple independent experiments. One-way ANOVA with Dunnett’s multiple comparisons test or the unpaired Student’s t test. ns, *p*>0.05, **p*<0.05, ***p*<0.01.


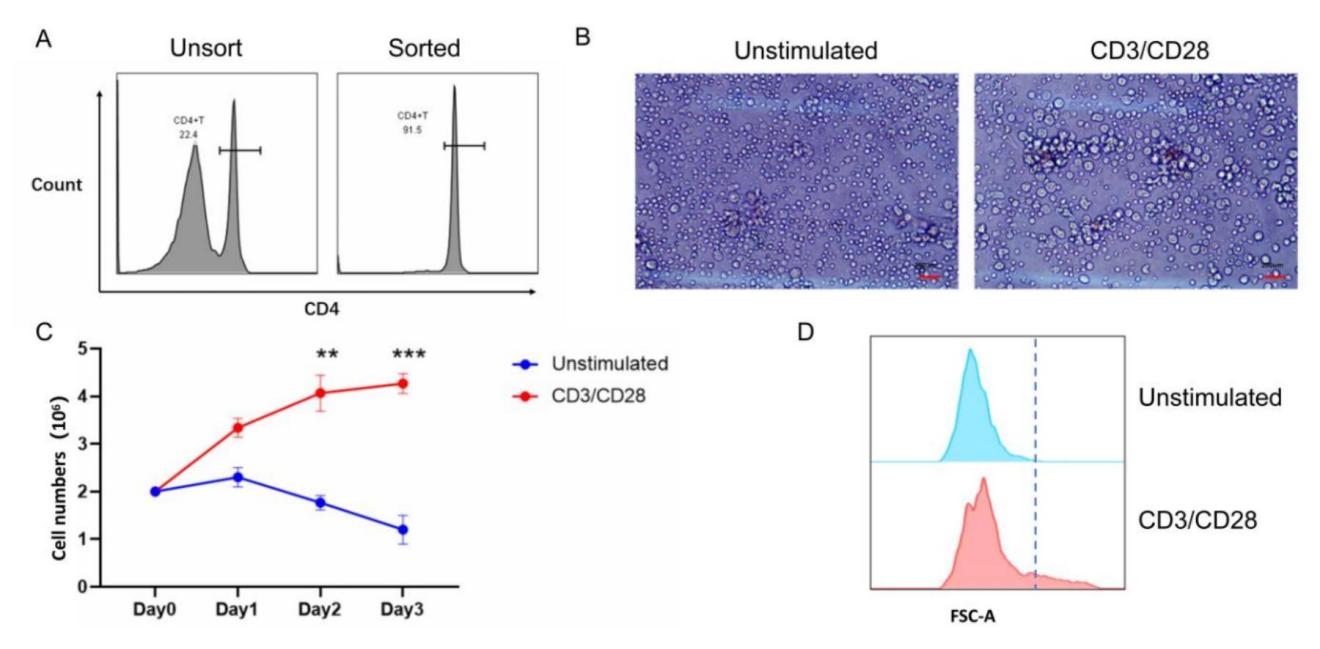


**Figure S7. Activation of CD4+ T cells.** (A) Flow cytometry was used to verify the purity of CD4+ T cells sorted by magnetic beads. (B) The morphology of activated and un-activated CD4+ T cells on the third day under an optical microscope at a scale of 200 μm. (C) The number of activated and un-activated CD4+ T cells over three days. (D) Flow cytometry was used to detect the size of activated and un-activated CD4+ T cells on the third day. The results were expressed as mean ± SD, ***p**<*0.01, ****p<*0.001, vs Unstimulated.


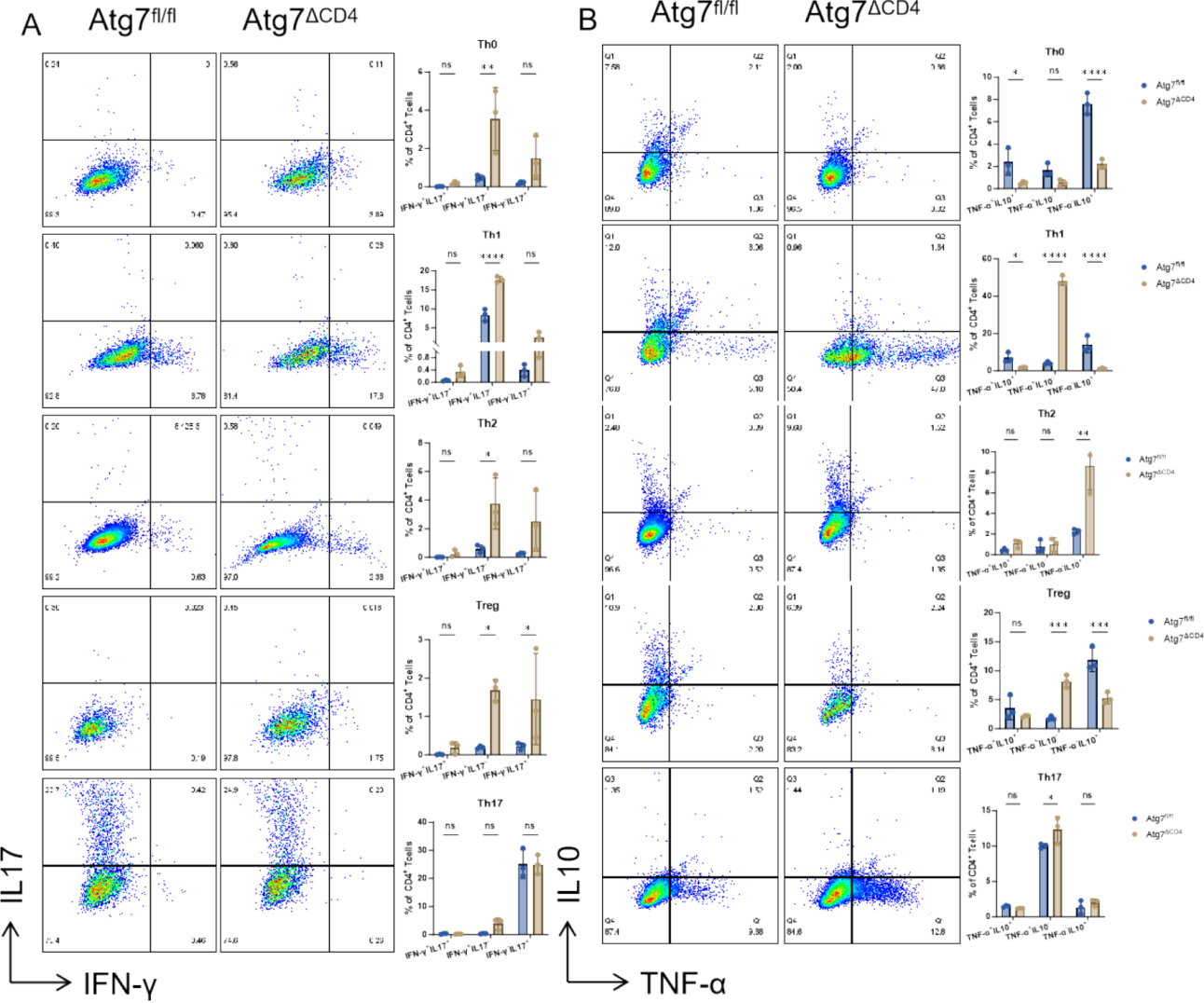


**Figure S8. Atg7 deficiency has opposing effects on Th1 and Treg cell differentiation.** (A-B) Detection of mouse CD4+ T cell differentiation using flow cytometry. Bars represent the mean ± SD from multiple independent experiments. Two-way ANOVA with Sidak's multiple comparisons test. ns, *p*>0.05, **p<*0.05, ***p <*0.01, ****p**<*0.001, *****p**<*0.0001.


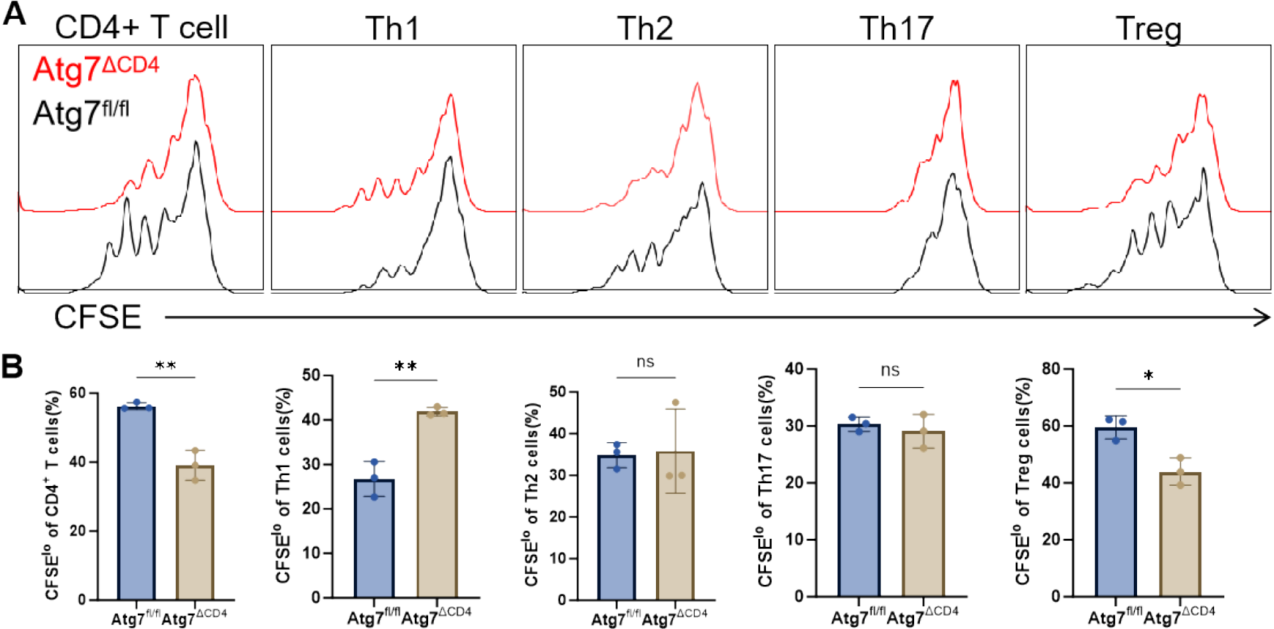


**Figure S9. The effect of Atg7 on the proliferation of CD4+ T cells and Th cells.** (A) Representative images of CFSE staining flow cytometry analysis. (B) Bar chart of the proportion of T cells with low CFSE (CFSElo) fluorescence intensity. Bars represent the mean ± SD from multiple independent experiments. Unpaired t test. ns, *p*>0.05, **p<*0.05, ***p <*0.01.


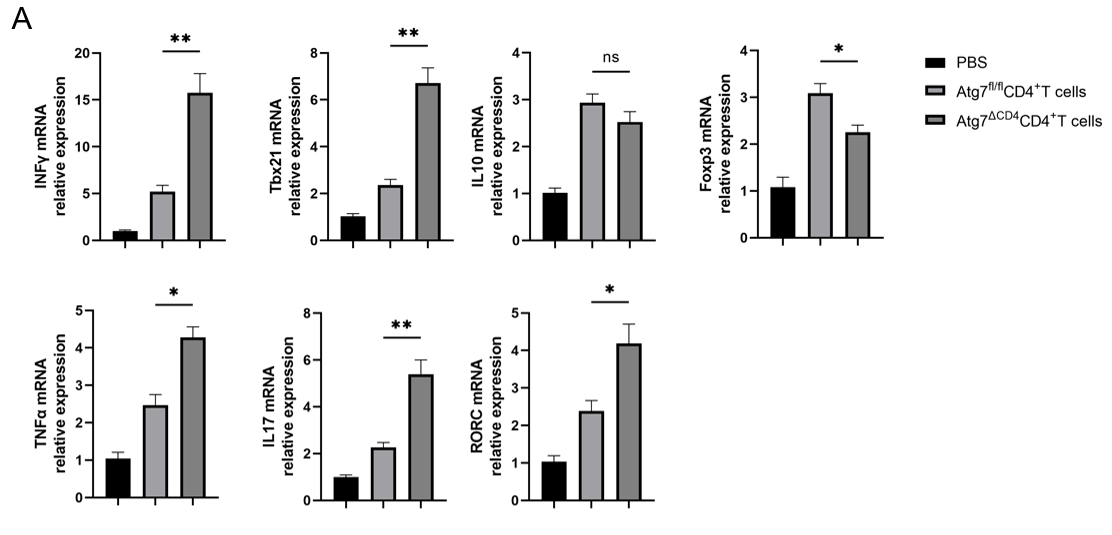


**Figure S10. Expression of** **mRNA of colonic inflammation-related factors after TNBS induction.** (A)qPCR is employed to detect mRNA expression level of INF-γ, Tbx21, IL10, Foxp3, TNFα, IL17a, and RORC in the lamina propria lymphocytes Extracted from the colon of TNBS-induced mice. n=6 in each group. Bars represent the mean ± SD from multiple independent experiments. Two-way ANOVA with Sidak’s multiple comparisons test. ns, *p*>0.05, **p<*0.05, ***p <*0.01.


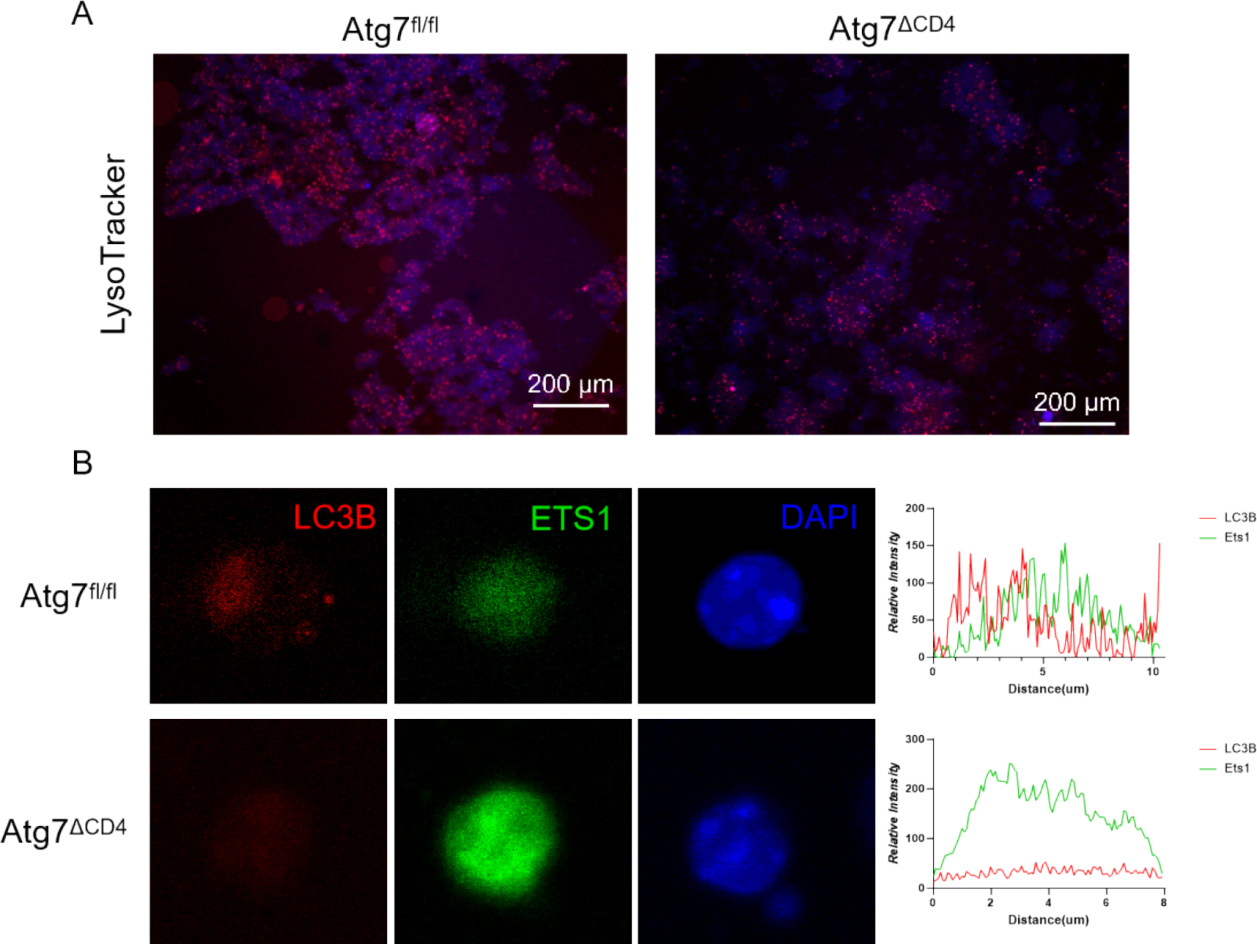


**Figure S11. Atg7 inhibits lysosomal function. (A)** Representative images of Lyso Tracker staining of naïve CD4+ T cells in the spleen of *Atg7fl/fl* mice and *Atg7ΔCD4* mice. Scale bars, 200µm. **(B)** Representative immunofluorescence images and co-localization analysis of the naïve CD4+ T cells of*Atg7fl/fl* mice and *Atg7ΔCD4* mice stained with LC3B (red) and ETS1 (green), and the cell nuclei were stained with DAPI (blue)**.**


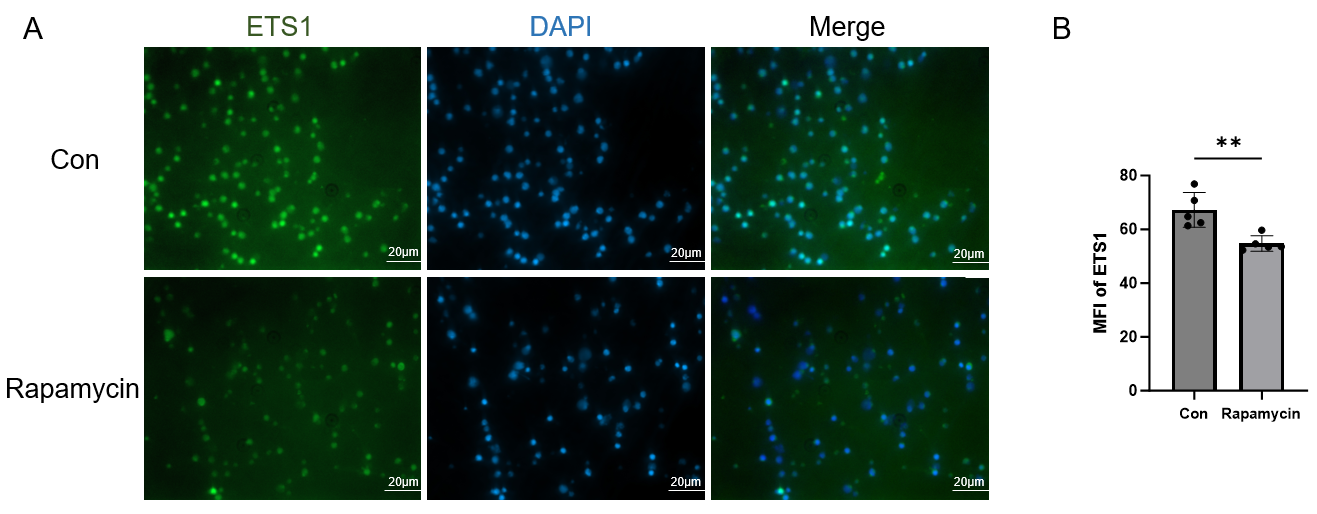


**Figure S12. Atg7 regulates the expression of Ets1 via autophagy.** (A) CD4+ cells are extracted from the lymph nodes and spleens of *Atg7ΔCD4* mice Subsequently, the cells are induced with or without 40 nM rapamycin for three days. On the third day, the cells are collected for a cell immunofluorescence assay. (B) Statistical diagram is the average fluorescence intensity obtained through statistical analysis using ImageJ. n=5 in each group. Bars represent the mean ± SD from multiple independent experiments. One-way ANOVA with Dunnett’s multiple comparisons test or the unpaired Student’s t test. ***p <*0.01.


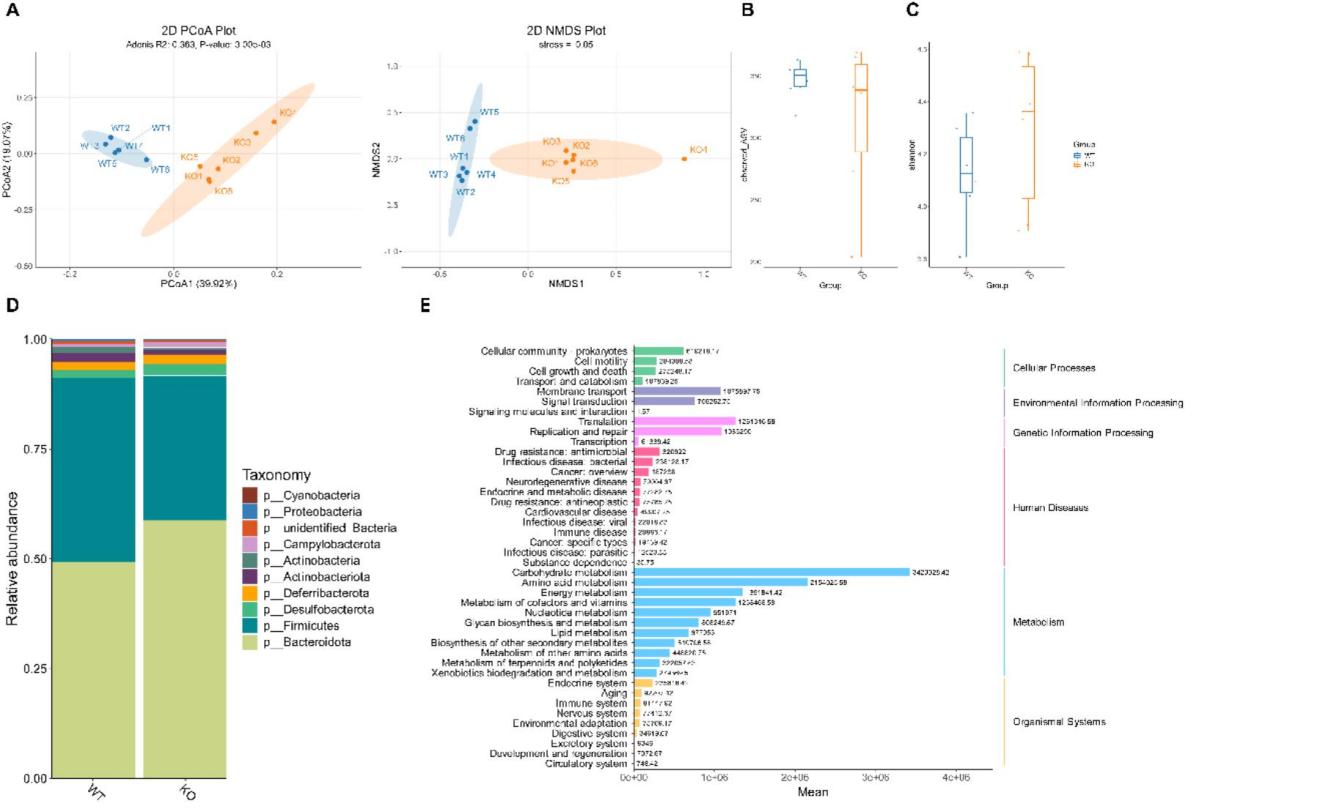


**Figure S13. The 16s rDNA sequencing experiment of mouse feces.** (A) Unweighted Unifrac distance PCoA analysis and NMDS analysis based on ASV. (B) Observed-species index. (C) Shannon index. (D)Stacked bar chart of relative abundance of species based on ASV. The vertical coordinate (Relative Abundance) represents the relative abundance. (E) Phylogenetic Investigation of Communities by Reconstruction of Unobserved States (PICRUSt2) secondary classification abundance bar chart based on ASV. WT: *Atg7fl/fl* mice, KO: *Atg7ΔCD4* mice. The differences between groups of the Alpha diversity index were analyzed by T-test and wilcox rank sum test.
